# QRxVision: Multimodal QR Encoding and Computer Vision for Domain-Specific Drug Discovery

**DOI:** 10.1021/acs.jcim.6c01321

**Published:** 2026-10-28

**Authors:** Mithony Keng, Kenneth M. Merz

**Affiliations:** † Department of Chemistry, 3078 Michigan State University, East Lansing, Michigan 48824, United States; ‡ Department of Biochemistry and Molecular Biology, Michigan State University, East Lansing, Michigan 48824, United States

## Abstract

Computational approaches applied to the drug discovery pipeline are invaluable because of their overall speed or time to solution, sustainability, accessibility combined with ever improving accuracy. Furthermore, the high-throughput virtual screening supports practical sampling of the vast chemical space for target-relevant bioactive compounds that can be experimentally validated further downstream in the drug development process. The current paradigm being practiced in computer-aided drug screening strategies centers around structure-based and ligand-based approaches supported by a range of computational tools including de novo design, molecular dynamics-based approaches, and machine learning (ML). Overall, the latter approach has shown great promise generating models of high computational speed coupled with good accuracy. In this work, we developed QRxVision, a domain-specific multimodal ML model explicitly optimized for kinase target assignment using similarity-based profile matching and retrieval. QRxVision utilizes image vision from a pretrained VGG16 (Visual Geometry Group–16) model for deep spatial feature extraction and cosine similarity calculation. By encoding drug multimodality-associated parameters (MAP) into standardized QR code images, QRxVision can evaluate the similarity between test and reference compounds. Drug targets can then be assigned via transitive profile matching. Validation using kinase inhibitors shows that QRxVision is 71% successful at assigning viable small molecules within the Top-5 predictions (*p*-value < 0.05). Overall, we believe that with the appropriate algorithmic modifications and domain retargeting, the QRxVision framework can be applied to other target assignment tasks.

## Graphical abstract

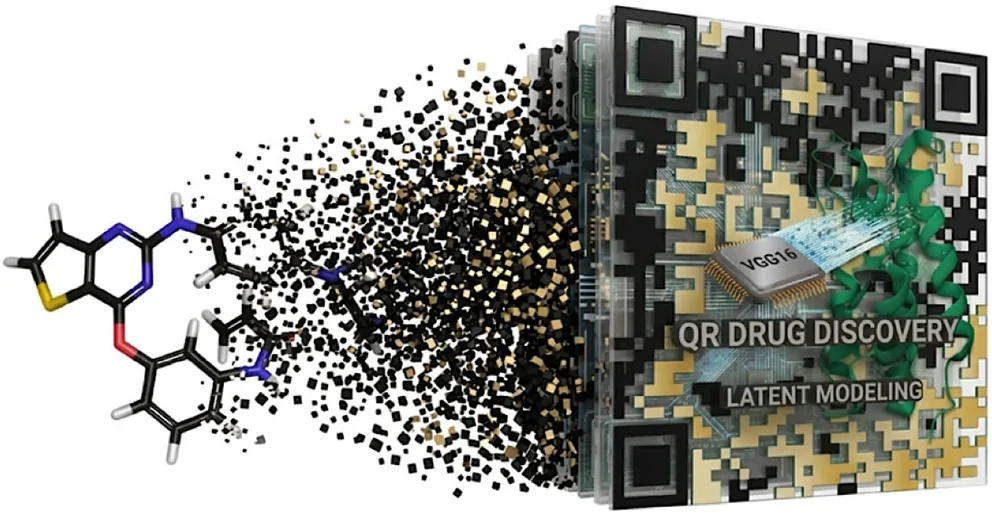

## Introduction

Drug discovery is a highly attractive field due to its impact on human health. Among the many disease targets, cancer represents the largest therapeutic area in the pharmaceutical industry, accounting for nearly 40% of the drug development market pipeline.^1,2^ This large resource engagement is justified considering that cancer (all types) is the second leading cause of death.^3^ Moreover, a majority of the research efforts are often clustered around modifying protein kinase activity either with a small molecule or an antibody-derived inhibitor.^4^ For example, molecular targets within the kinome by clinical trial volume are highly concentrated around EGFR and HER2 since breast cancer is a leading oncological diagnosis.^5,6^ Trends in the occurrence of all cancers have significantly risen over the last decades,^7^ and thus, there is a highly motivated endeavor to develop improved methods for discovery of new bioactive compounds that are specifically relevant to the kinome.^8^

With the recent jump in AI capabilities, novel machine learning engines have been added to the computer-aided drug design toolkit.^9^ ML models have notable capabilities including low barriers to execution, scalability, transferability, and high throughput.^10,11^ Within the kinase-related research, computational frameworks have increasingly been deployed to navigate the massive search space for candidate compounds and map prospective small-molecule interactions across the kinome. However, a fundamental challenge remains in kinase target assignment and drug-target profiling; traditional frameworks are heavily reliant on single-modality representations. Just as deep structural modeling architectures can characterize target proteins, virtual screening workflows require architectures capable of unifying diverse data modalities.

For kinase target matching and profiling, candidates are traditionally evaluated by standard machine learning algorithms such as transformer, random forest, support vector machines, and/or neural networks.^12–14^ Popular open-source models like ChemBERTa^15^ (Transformer) and SMILES2Vec^16^ (recurrent neural networks) can efficiently predict bioactivity and chemical/physical properties from SMILES strings using self-supervised and supervised learning approaches, respectively. Despite their efficiency, these 1D serialization methods and graph-based approaches encounter a distinct methodological gap: they struggle to cleanly integrate heterogeneous descriptors from drug and target properties within a transitive matching framework. Furthermore, standard shallow networks fail to extract the high-dimensional, nonlinear spatial dependencies required for precise profile retrieval, particularly when constrained by data-scarce scenarios. Furthermore, multimodal feature integration has been found to improve drug-target interaction and drug property predictions by approximately 5% and 4%, respectively, over unimodal baselines.^17^ Alternatively, ChemVAE encodes SMILES into an implicit 2D input for de novo drug design using the variational autoencoder method; however, the lack of true intrinsic geometric parameters and target awareness often results in inactive small molecules or synthetically inaccessible compounds.

More recently, model training regimens for bioactivity have shifted from relying disproportionately on descriptors originating from 1D SMILES to a 2D representation for topological rendering and improved latent space mapping. Chemception^18^ generates 2D molecular images from input SMILES for ligand-based chemotyping using convolutional neural networks (CNNs). Despite better spatial information, both ChemBERTa and ChemVAE^19^ generally outperform Chemception. This performance discrepancy may stem from image noise degrading the quality of the latent representation. Advanced self-supervised frameworks like ImageMol^20^ counter this issue by leveraging a CNN backbone pretrained on roughly 10 million 2D molecular images with robust data augmentation. By effectively suppressing noise interference and improving latent representations, ImageMol has been shown to deliver superior performance compared to earlier models. Despite this favorable performance, ImageMol is computationally heavy and requires millions of training images to remain effective. This leaves a critical gap for specialized tasks such as kinase target profile retrieval, where experimentally validated data sets are limited and high structural homology creates highly clustered data points that are difficult to differentiate.

In order to help improve upon drug kinase assignment throughput and precision, additional ligand-related features and target-relevant modalities will need to be added to the input data. Herein, we propose transforming drug multimodality-associated parameters (MAP) into synthetic 2D images to leverage computer vision for high-dimensional feature extraction. Moreover, to speed up the computer vision task, the MAP data need to be imaged in a manner that moves beyond traditional molecular graphs or molecular images. In this work, we introduce and validate a proof-of-concept, domain-specific model called QRxVision that employs QR code technology, computer vision, transfer learning, and transitive relationships for effective drug-kinase target assignment. Figure 1 shows the four hallmark steps (QR sampling → latent feature extraction → vector similarity calculation → transitive matching) for QRxVision that are executed computationally for kinase inhibitor identification. The advantages of a QR code based approach are data storage capacity, space efficacy, built-in error correction, and global interoperability (standardized). Although QR code technology has been used prior to encode topological information to predict chemical properties,^21^ to the best of our knowledge, it has never been implemented in as described herein and for the intended task of drug-target assignment.

**Figure 1:**
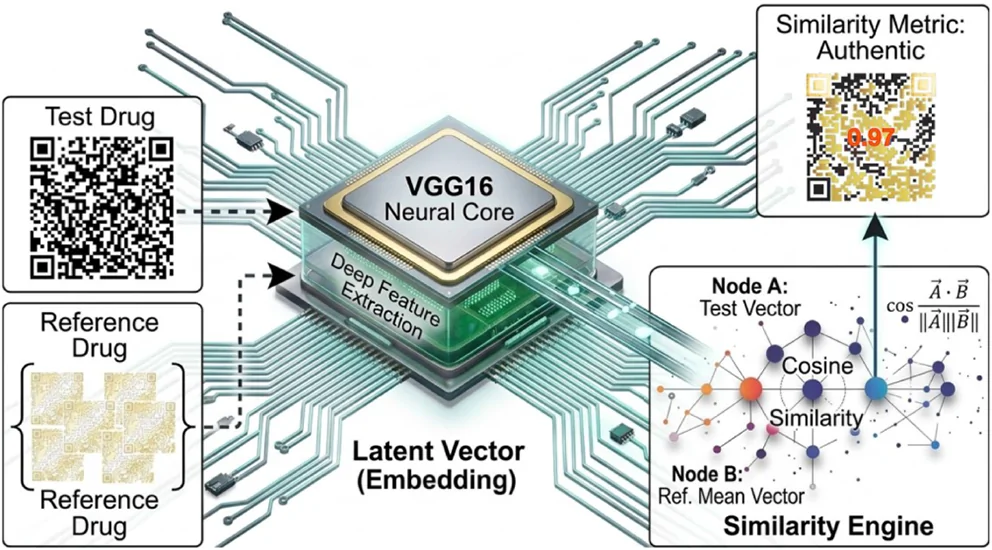
QRxVision programmatic steps that initiate with MAP embedded QR codes, feature extraction using a pretrained image classifier, feature vector similarity calculation, and target assignment using similarity score and transitive matching.

It is critical to clarify that QRxVision is not designed as a foundational screening platform, which typically requires millions of training instances. Instead, this work serves as a specialized proof of concept tailored for domain-specific target retrieval. By validating the framework on a highly curated data set of 111 reference compounds and 35 test samples with experimentally verified kinase bioactivity, we demonstrate that QRxVision offers a lean approach for target assignment in sparse chemical environments where broad deep learning models can succumb to overfitting.

## Computational Methods

The computational methodology of QRxVision evaluates candidate small molecules against the human kinome using a sequential four-step pipeline that consists of multimodality data curation, visual matrix mapping, latent feature extraction, and transitive profile matching. Transforming abstract chemical-biological properties into a stable visual coordinate system provides the foundational rationale for this specific sequence. By projecting heterogeneous descriptors into a standardized image format, the workflow bypasses data-intensive graph simulations in favor of deterministic image recognition. This structural arrangement ensures that subsequent mathematical steps—ranging from descriptor calculation to vector alignment—operate within a unified, reproducible projection space.

### Validated Drug and Target Binding Site Data

Kinase inhibitors and their corresponding target binding site information were retrieved from the Therapeutic Target Database^22^ (TDD). Both test and reference drug compounds that are specified for kinases and cancer indications were identified by querying the approved drugs module. The resulting search produced a total reference data set of 111 kinase inhibitors and related compounds. The names of the reference compounds are tabulated in Table S1 in Supporting Information. Information about binding site amino acid quality and frequency in binding was obtained by accessing the target detail module for a given queried drug. For each amino acid type, we counted the number of times it interacted with a drug. The residue-drug interaction distance was defined as <5Å,^23^ which also includes hydrogen bonding interactions (<3.5Å).^24^

### Molecular Descriptors Generation for MAP Feature Encoding

Drug compound molecular descriptors calculated using the RDkit Chem library includes topological surface area (TPSA), van der Waals surface area (VSA), logP, ring count, double bond count, triple bond count, positive charge count, and negative charge count. Additional required descriptors are SMILES, heteroatom count, halogen count, radii mass, and electronic. Both radii mass (S) and electronic (E) are ad hoc descriptors for steric and electronic fingerprinting, respectively, and can be calculated with eq 1 and eq 2, respectively.

1 $$S=\frac{n\overset{n}{\underset{i=1}{\sum }}r_{i}}{\overset{n}{\underset{i=1}{\sum }}m_{i}}$$

2 $$E=\frac{\overset{n}{\underset{i=1}{\sum }}{Ι}_{i}\overset{n}{\underset{i=1}{\sum }}\chi_{i}}{n\overset{n}{\underset{i=1}{\sum }}\alpha_{i}}$$

where *i* is the *i* ^th^ atom, *r* is the atom radius, *m* is atomic mass, I is the ionization energy, χ is the electronegativity, α is the polarizability, and *n* is the total atoms of a system.

To develop the target-relevant descriptor, we quantified the amino acids within the kinase binding site that directly interact with the ligand (validate kinase inhibitor). The target feature vector consists of 20 columns, each representing a specific amino acid type. The input value corresponds to the frequency of interactions between an amino acid and a reference drug; for instance, an amino acid type having a frequency of three interactions is assigned a value of 3, while noninteracting types are assigned 0.

### Test and Reference Drug MAP Feature Vector

The data structure for a reference drug and a test drug follow the scheme displayed in Figure 2. For a reference drug, we combined the drug features and target-relevant (kinase) features into a single row of the feature vector representing that reference drug data point. There are 111 data points for the 111 total reference drugs in this work. Each data point in the sponsor matrix is mapped into a low-dimensional embedding (QR code). For a test drug, we replace the reference drugs feature vectors in the sponsor matrix with the feature vector of the single test drug, replicated across all 111 rows of data points. The target-relevant features from the original matrix are retained, forming a hybrid matrix that transitively represents the test drug’s potential interactions across the known kinase targets. This is feasible because each row of the matrix has a particular kinase designation (i.e., label). Similarly, each data point of the hybrid matrix (data points belonging to a single test drug) is embedded onto a QR code.

**Figure 2:**
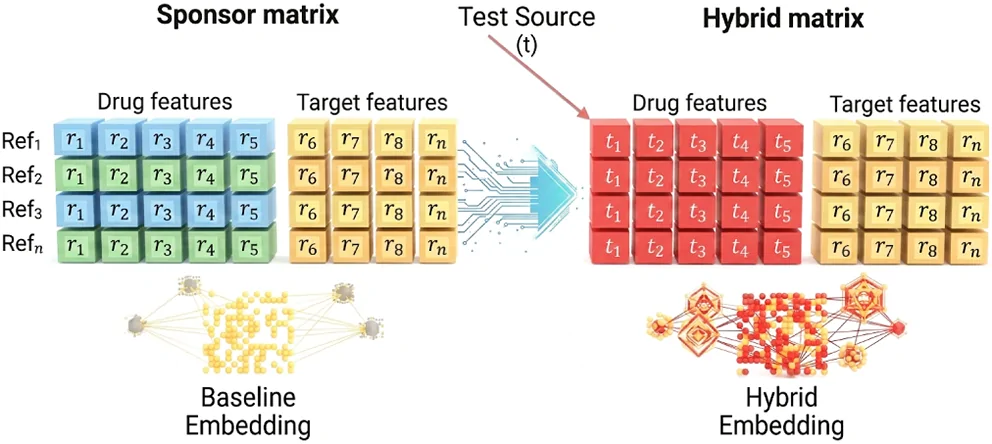
QRxVision reference and test input data setup. For test input data, the reference drugs features (alternating blue and green) are substituted with the test drug features (red) replicated across 111 rows and joined with the target features (alternating yellow and orange) of the initial reference drugs. This is reference feature adoption and constraint. Each row of data points is embedded to form a QR code image for high depth dimension feature extract for cosine similarity calculation.

### QR Code Generation Process

There are three main interpretable steps in the QR code generation: vector serialization → matrix mapping → image rendering. For vector serialization, *V* = [*x*_1_,*x*_2_, ..., *x*_n_] is a vector of extracted feature data for a drug row. To structure the data in a vector, we added an * (asterisk) delimiter between each feature to create a string transformation $S={⊕}_{i=1}^{n}\left(x_{i},\;*\;...\;*\;,x_{n}\right)$ where ⊕ represents the string concatenation of elements. Next, the QR matrix mapping *S* of to a 2D binary matrix *M* uses an encoding function *f* : *S* → *M*_*i*_ ∈ {0,1}^*m*×*m*^, where *m* = 290 is the dimensions of the QR grid. More precisely, the encoding function *f*(*S*, *V*, *EC*) processes three key inputs: the transformed string (*S*), data fitted grid version (*V*), and the Reed–Solomon error correction (*EC*). Finally, the image rendering uses a function *P*(*M*_*i*,*j*,*k*_) for geometric transformation where each binary element (*i*,*j*) in matrix *M* results in a white pixel block *M*_*i*,*j*,_ *_k_* = 0 if and black if *M_i_*,_*j*,_ *_k_=* 1.

It is worth noting that the algorithms indicated here are designed to maximize visual variance, meaning a minor change to an input character will drastically alter the spatial arrangement of the resulting matrix. In a standard supervised network training to regress continuous global molecular properties, this high visual variance would introduce disruptive spatial noise. However, QRxVision operates strictly as a deterministic similarity-matching engine. Since our encoding function *f*(*S*, *V*, *EC*) enforces a standardized descriptor sequence and a fixed pixel dimensions layout (M), every molecular data point undergoes the exact same geometric projection. This fixed visual scrambling acts as a reproducible, nonlinear projection space. Local feature modifications yield highly consistent, traceable alterations in edge density and line continuity. The early convolutional layers of the pretrained VGG16 backbone capture these precise structural shifts, mapping them into a coherent latent trajectory that preserves the relative distances between related compound-target profiles

### Input Data Augmentation

Following standard image classification best practices, we augmented the input canonical SMILES with five permutations which produce the same identical compounds; however, they generate slightly different patterns for the five QR codes (see Figure 3). We used the same python code to generate these QR codes as we did for the canonical SMILES. Furthermore, the importance of the data augmentation step is to promote QRxVision’s robustness by reducing overfitting, stabilizing prediction accuracy and lowering feature or scaffold bias. We did not accommodate any other common augmentation techniques like image perspective warping, noise/blur introduction, or random erasing because canonical QR codes are globally consistent through the practice of standardization. Additionally, our model uses transfer learning without trainable parameters for feature extraction, so the risk of overfitting is very limited. The performance of the augmented test set was compared to the test set without augmentation.

**Figure 3:**
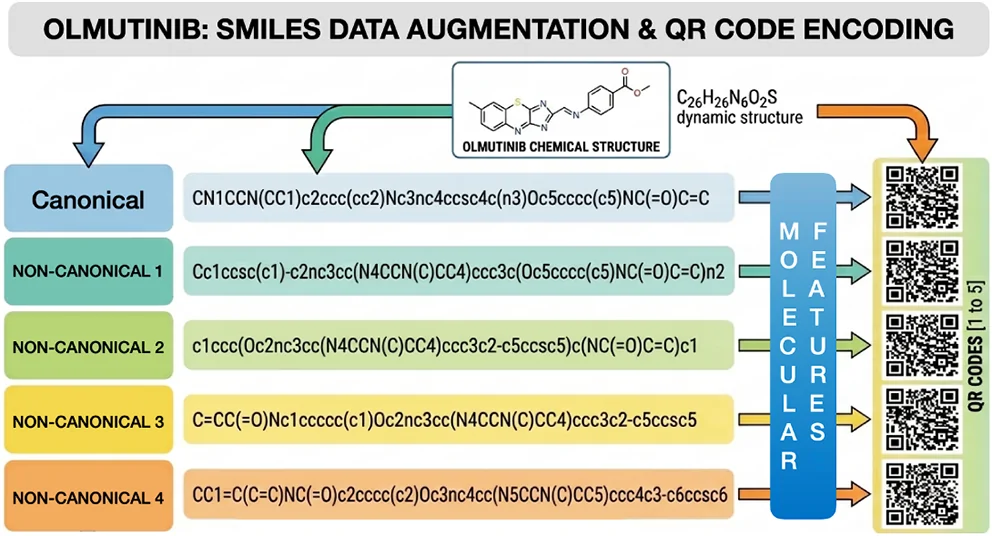
SMILES augmentation scheme to generate subtle variation of input QR code images to condition VGG16 model latent space invariance for a given compound (i.e., Olmutinib). For this example, the augmentation is 5-fold (five variants of the canonical SMILES are produced).

### Establishing VGG16 Optimal Operating Architecture

VGG16 contains six blocks of CNN layers and a classifier block. Blocks 1–2 each contain two CNN layers and a max pooling layer, transforming the initial QR code image dimensions (224 × 224 × 3) to 112 × 112 × 64 and 56 × 56 × 128 feature map, respectively. Blocks 3 through 5 each contains three CNN layers and a max pooling layer, ultimately resulting in a feature map size of 7 × 7 x 512.

Since we are utilizing transfer learning to extract the 1D feature vector from the QR code, the pretrained classifier (last block containing a flatten layer and three fully connected dense layers) was removed. In its place, we used a global max pooling (GMP) layer to convert 3D feature maps from Blocks 1–5 to a 1D feature vector for cosine similarity calculations. To determine which blocks produce the best performing feature map representation we carried out an ablation study using layer truncation. Generally, Blocks 1–3 extracts low-level features such as edges, lines, colors, and simple geometric patterns, and since these qualities are highly relevant to QR codes, we truncated the network at Block 4 or Block 5. Truncating Block 4 means the output from Block 4 undergoes global pooling to create a 1D feature vector; truncation at Block 5 uses the output from Block 5. Next, we evaluated the performances of the two global pooling types—max and average—using the optimized VGG16 CNN architecture.

### VGG16 Performance Benchmarking

For performance qualification testing, we evaluated the optimized VGG16 model against DenseNet121 and MobileNet against a feature extraction task. All three image vision models have been pretrained on the same ∼1.28 million images from the ImageNet-1K data set. There are approximately 139, 8, and 4.2 million parameters for VGG16, DenseNet121, and MobileNet, respectively. For all three vision models, the parameters were left at their default setting, but their classifiers were removed for transfer learning. Again, classifier removal is necessary to retain the latent feature vector representation and salvage the numerical fingerprint of the QR code. As shown in Figure 4, we mirrored the different architectures of DenseNet121 and MobileNet to the optimized VGG16 model to make a fair comparison. The max pooling step resulted in 1D feature vectors of size 512, 1024, and 1024 for VGG16, DenseNet121, and MobileNet, respectively. Similarity scores for the 1D feature vectors generated by the three image models for the test set and the reference set were calculated using the cosine similarity method from the Scikit-learn library in python. For Benchmarking, we tested 35 test compounds (confirmed to have binding activity with one or more kinases) against 35 reference inhibitors on 45 target classes (number of unique kinase groups after family and isoform merging). For the complete list of test compounds and target kinases, see Table S2 in Supporting Information.

**Figure 4:**
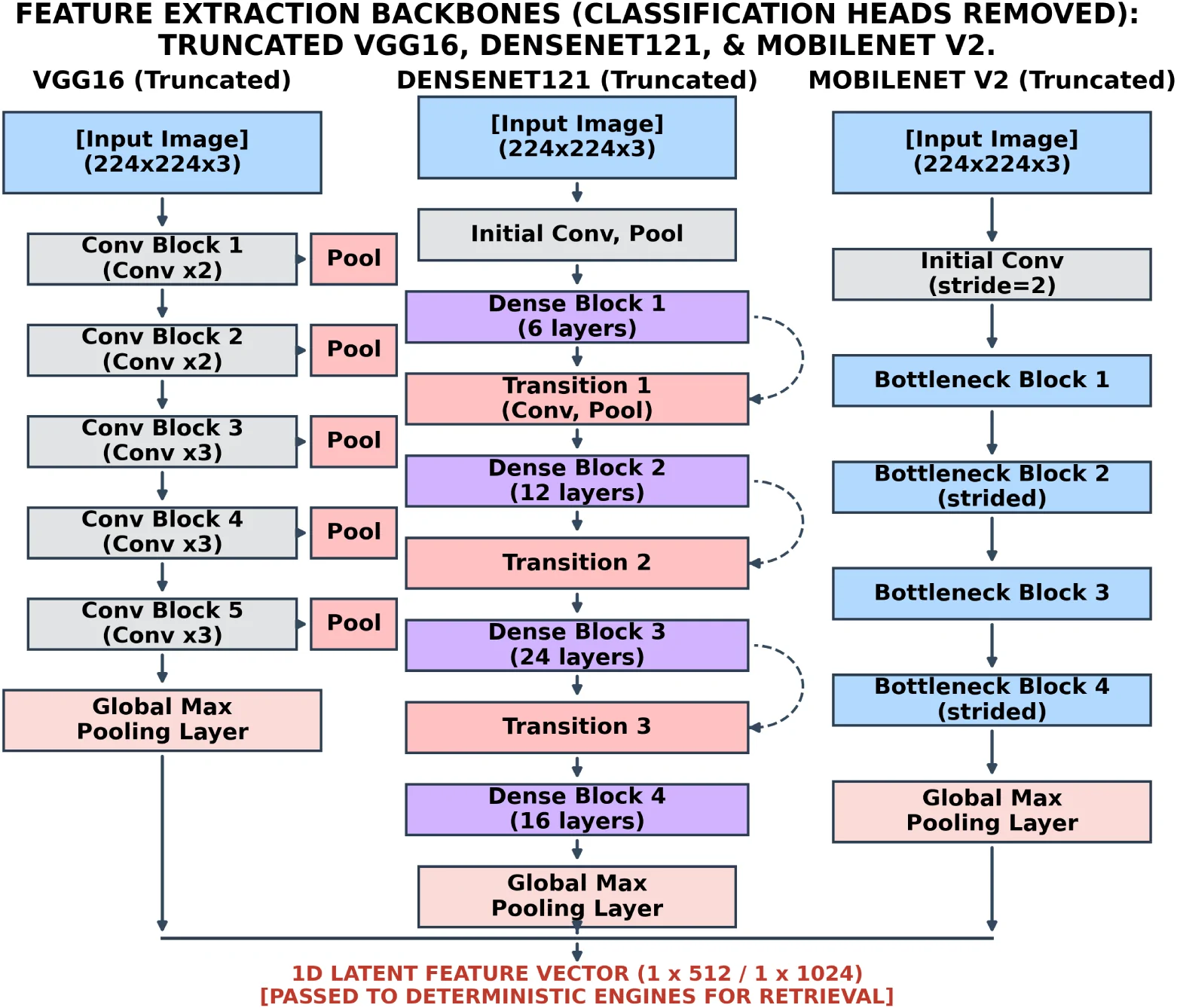
Architectures of three image vision models VGG16, DenseNet121, and MobileNet. The arrow represents the flow of 3D latent feature vectors (feature map) going from higher to lower spatial size. The “include_top = False” designation in our code indicates classifier removal. The max pooling consolidates highest vector values from spatial dimensions to depth channels to produce output feature vector of size (512, 1024, or 1024) 1D, respectively. Both DenseNet121 and MobileNet are used to benchmark VGG16.

### Cheminformatics Baselines Evaluation

To evaluate the QRxVision multimodality and image-embedding implementations, we utilized three baseline architectures on a data set of 106 samples. For Baseline 1 (Morgan fingerprint + native drug descriptors + native target descriptors) configuration, drug SMILES were converted to the 1024-bit ECFP4 (Extended-Connectivity Fingerprints) with a bond radius of 2 using RDKit. The vector containing the extracted Morgan fingerprints was then combined (early fusion) with our 12 QRxVision native drug descriptors to form a raw, numerical drug feature matrix, Baseline 2 (Morgan fingerprint + native targets) was implemented by combining the 1024-bit ECFP4 drug fingerprints with our native 20 amino acid frequency descriptors into a drug-target fusion numerical matrix. Finally, Baseline 3 (Native drugs + native targets) uses the QRxVision raw drug-target descriptors (early fusion) without the QR embedding.

To assign nearest-neighbors for Baseline 1 and Baseline 2, individual cosine-similarity matrices were calculated independently for drug descriptors (*M*_*drug*_) and target descriptors (*M_target_*). These were then combined in a late-stage score fusion to derive interaction space using an elementwise Hadamard product (eq 3):

3 $$H=M_{drug}⊙M_{target}$$

where H is the similarity product from the multiplication of the *M*_*drug*_ drug similarity matrix and *M_target_* target similarity matrix. Alternatively, for Baseline 3, the drug and target descriptors underwent early stage feature fusion to mimic QRxVision (minus the QR code embedding). The final cosine similarity was then calculated across this joint drug-target vector space.

Since QRxVision Top-5 ranking metric considers off-target, primary, and secondary binding relationship as positive hits, the same constraints were given to the ranking protocol for these baseline through a promiscuity-relaxed evaluation loop. To carry this out, we mapped every unique compound SMILES across the entire data set to its total set of experimentally known viable kinase targets as defined in eq 4:

4 $$H_{relax}\left(s_{i}\right)=\left\{t\in \mathrm{Dataset}|\mathrm{Viable}\left(s_{i},t\right)=\mathrm{True}\right\}$$

where $H_{relax}\left(s_{i}\right)$ represents the set of viable targets for compound *s*_*i*_ under a relaxed constraint, *t* denotes a specific kinase target, and *s* is the specific drug compound. To verify the Top-5 matching performance, a retrieval task is evaluated as correct according to the indicator rule defined in eq 5:

5 $$\mathrm{Results}\left(i\right)=\{\begin{array}{l} 1,,\mathrm{if}\left\{{\mathrm{Predicted Target}}_{1...5}\right\}\cap H_{relax}\left(s_{i}\right)\neq \phi \\ 0,,\mathrm{otherwise} \end{array}$$

where a hit (Results­(*i*) = 1) is achieved if the intersection operator (∩≠ϕ) identifies any overlap between the Top-5 predicted targets and the set of true targets.

## Results and Discussion

QRxVision utilizes a transfer learning framework,^25,26^ employing a pretrained VGG16 architecture to perform fixed-feature extraction from molecular properties encoded as QR images.^28,29^ By mapping molecules into high-dimensional latent space, our self-supervised model identifies functional similarities through cosine similarity calculations rather than traditional supervised classification. This approach effectively addresses the data limitations common in kinase research (e.g., supervised GNNS that require massive data sets), enabling target assignment via transitive matching against known reference inhibitors (or relevant kinase binding compounds) without the need for task-specific retraining. For this work, we define a test drug and kinase match as a pair that forms viable binding interactions, which means that a weak, strong, off-target, primary, and secondary binding relationship are all classified as positive hits. Moreover, we have conducted extensive validation and benchmarking to ensure that the input data representation style and the VGG16 architecture are optimized to achieve robust performance and reliable target assignments.

### Optimized VGG16 CNN Architecture

For the CNN truncation and global pooling ablation studies, VGG16 hyperparameters were maintained at their default pretrained values. The ablation tests utilized 1D feature vectors extracted from Block 4 and Block 5, both employing a GMP scheme. As previously alluded to, Block 4 feature maps retain more local geometric detail, whereas Block 5 provides a more global, abstract representation of the original QR image. While Block 5 is typically preferred for general object recognition and classification in transfer learning, it often suffers from spatial resolution loss from more repeated pooling. Being that our QR image is stylized in a geometric format, we also selected Block 4 to see if we can preserve the more refined local features and precise pixel correlations necessary for good pattern recognition.

The performance results for Block 4 and Block 5 are shown in Figure 5A. The statistical metric that we use to differentiate performances is the Top-*k* accuracy because it is effective for subtle class differences across the structural continuum of kinases. For 45 different classes of kinases on a test batch of 35 compounds, Top-1 accuracy for Block 4 has nearly double the target assignment accuracy than Block 5. Although Block 4 has the better precision (mode = 1 vs mode = 2), from K = 2 to K = 5 Block 5 performed better which leads to an overall higher accuracy (71.4% vs 65.6%). Both Block 4 and Block 5 failed to assign the correct target kinases for 12 (34.2% false negatives) and 10 (28.6% false negatives) compounds in the test set, respectively. Furthermore, our estimated area under the cumulative match characteristic (AU-CMC) is 0.80 (Block 4) and 0.82 (Block 5), which is competitive with other models.^20,27,28^ To put things into perspective, the typical AUROC (area under the receiver operating characteristic curve) for ML drug discovery ranges between 0.70 to 0.99 for high-performing and state-of-the-art models,^29^ with specific kinase image-based models^30^ achieving an AUROC of ∼0.86 for target discovery. Although we evaluated QRxVision using a retrieval space metric (Top-*k*) for multicandidate ranking, our AU-CMC score of 0.82 across 45 target classes aligns well with these established AUROC baselines.

**Figure 5:**
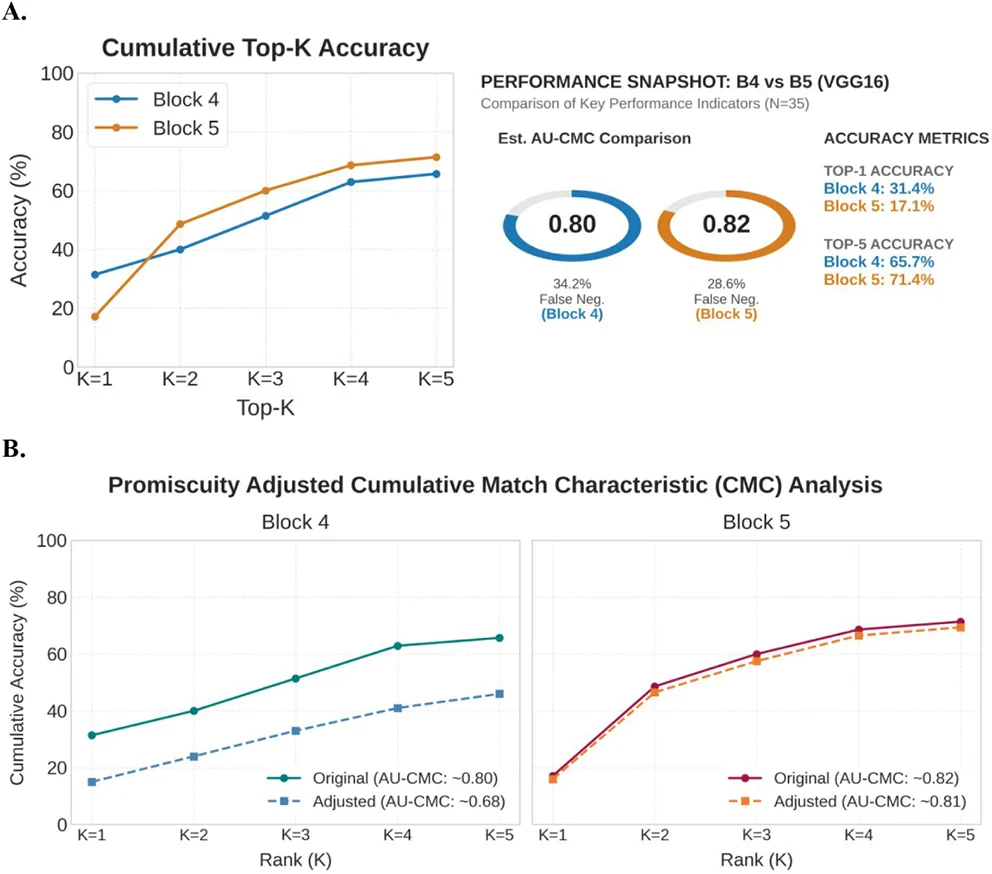
Performances comparison of VGG16 CNN Block 4 and Block 5 feature vector outputs. A) Block 4 outperforms Block 5 in Top-1 correct assignments. However, from Top-2 through Top-5, Block 5 performance surpasses Block 4, resulting in higher overall accuracy and lower false negative rates (higher CMC). B) Comparison of CMC changes for Block 4 and Block 5 following the removal of the promiscuous binder staurosporine. Solid and dashed lines represent the CMC with and without staurosporine, respectively. The minimal change in CMC after removing these effects suggests Block 5 is more robust than Block 4.

Since our kinase assignment utilizes transitive matching, the quality of the reference compound significantly impacts the integrity of QRxVision’s performance. We report that 20% (7 of 35) of the reference for Block 4 assignments and ∼9% (3 of 35) for Block 5 are through staurosporine, a well-documented promiscuous kinase binder. Staurosporine has a broad affinity within the kinome, binding to as high as 90% of the human kinases that have been screened.^31^ For our study, the kinases sponsored through staurosporine for assignment included EGFR, HER2, BCR/ABL, PI3Kα, JAK, MEK, KIT, PDGFRα, and AKT. To correct for the likely negative effect on our performance metrics, we removed the test compounds that were sponsored by staurosporine for both Block 4 and Block 5. The resulting adjusted accuracy values are 57.1% (16 of 28) for Block 4 and 68.8% (22 of 32) for Block 5. The adjusted AU-CMC for Block 5 (0.81) dropped less than Block 4 (0.68), indicating better robustness since its performance remained more stable after the change in the test samples (see Figure 5B).

### GAP and GMP Feature Vector Representation Qualities

Next, we tested the performance of the alternative global average pooling (GAP) scheme against our arbitrary GMP baseline using exclusively the Block 5 CNN architecture moving forward. For the global pooling test set, we added back in the compounds that were removed for staurosporine. This is acceptable since it had minimal impact on Block 5, and it is necessary for a fair ablation assessment of the two pooling schemes. Interestingly, results for Block 5 with GAP only generated two kinase assignments that have staurosporine as the transitive matching sponsor (see Figure 6A). Comparatively, GAP has the better Top-1 precision, with a ∼28.6% (MRR = 0.428) success rate within the first attempt, whereas GMP has the better overall accuracy with a Top-5 success rate of ∼71.4%. In addition to a higher overall AU-CMC for GMP than GAP (0.818 vs 0.753), we find a lower miss rate (∼28.6 vs ∼34.3%) to be particularly valuable for recall maximization in early stage drug candidate discovery.^32,33^ Moreover, to determine whether GMP has a significantly lower miss rate than GAP we used the two-sample proportion Z-test to obtain a *p*-value of ∼0.298, which is greater than the 0.05 significant threshold. Simply put, there is a ∼29.8% probability that the lower miss rate for GMP occurred by chance and not due to better latent representation output.

**Figure 6:**
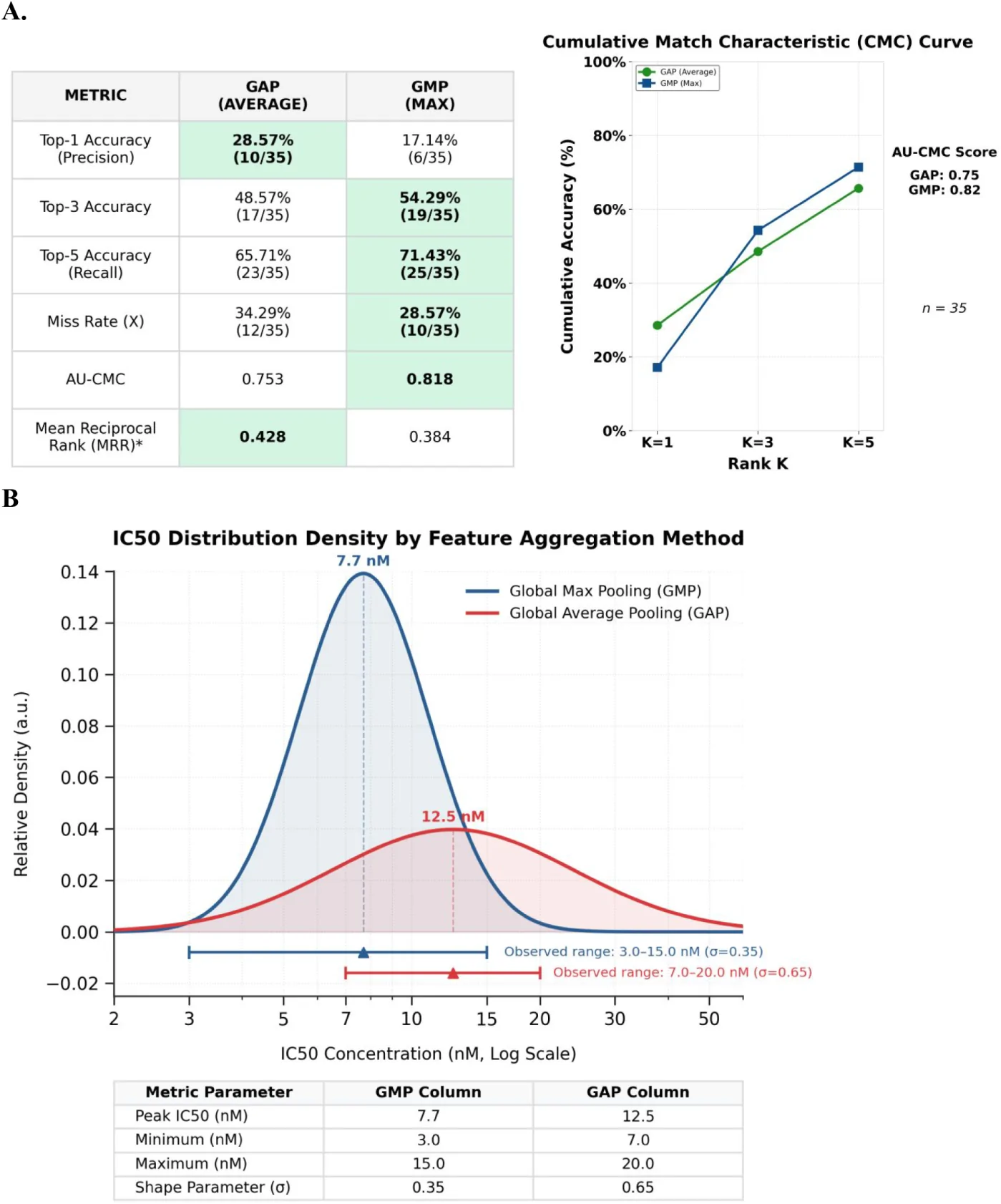
Performance comparison of feature vector generated via Global Average Pooling (GAP) and Global Max Pooling (GMP). A) GAP achieves higher Top-1 precision, whereas GMP outperforms GAP from K = 2 to K = 5, resulting in superior overall accuracy. Base on Top-K performance, GMP (blue) has a slightly higher AU-CMC by capturing more true positives to within K = 5 than GAP (green), leading to fewer Top-5 misses. B) Comparative IC50 analysis shows that GMP (blue) is more precise at capturing lower IC50 assignments than GAP (red).

We then cross-referenced the sponsor-kinase pairs assigned by GAP and GMP against databases like Pubchem, ChEMBL, and BindingDB to collect information on their *IC*_50_ values, if available. We reason that the more faithful latent representations lead to better sponsor-kinase pairs which translate to stronger binding interactions, and this can be observed through the *IC*_50_ values. We infer from the estimated *IC*_50_ distribution profiles that GMP translated to stronger binding pair matches with peak frequency (mode) *IC*_50_ of 7.7 compared to 12.5 for GAP for our test set (see Figure 6B). Despite achieving better potency pairing for GMP, the difference between the two *IC*_50_ values are not statistically significant (*p =* 0.06), although one can argue that it is marginally significant. While the miss rate (*p* = 0.298) and binding potency (*p* = 0.06) are not statistically significant at the 95% confidence level; however, the consistent outperformance of GMP in relevant practical areas suggests to us a robust advantage in its favor.

### Optimized VGG16 Model Transfer Learning Performance Differentiation

To ensure that VGG16 was optimized to the standard expected for QRxVision, we benchmarked it against the two equally prominent vision models DenseNet121 and MobileNetV2. For testing, we sampled 27 kinase inhibitors from the original test batch containing 35 compounds. It is worth mentioning that DenseNet121 and MobileNetV2 are ∼3-fold and ∼30-fold faster than VGG16, giving them a throughput advantage. Conversely, we report in Figure 7A that our optimized VGG16 outperforms its DenseNet121 and MobileNetV2 counterparts in metrics that include precision, sensitivity, F1-score, and error rate. Although VGG16 has the overall highest Top-5 accuracy (81.5%), DenseNet121 Top-1 accuracy (∼33.3%) is nearly twice as good. According to the *p*-values (*p* < 0.001) obtained from the one-sample binomial test, all three models offer a statistical advantage over random target assignments, as indicated by the knowledge gap (i.e., learning effect) from the baseline in Figure 7B. Furthermore, VGG16’s performance gain was statistically significant compared to DenseNet121 (*p*<0.05), though not significantly different from MobileNetV2.

**Figure 7:**
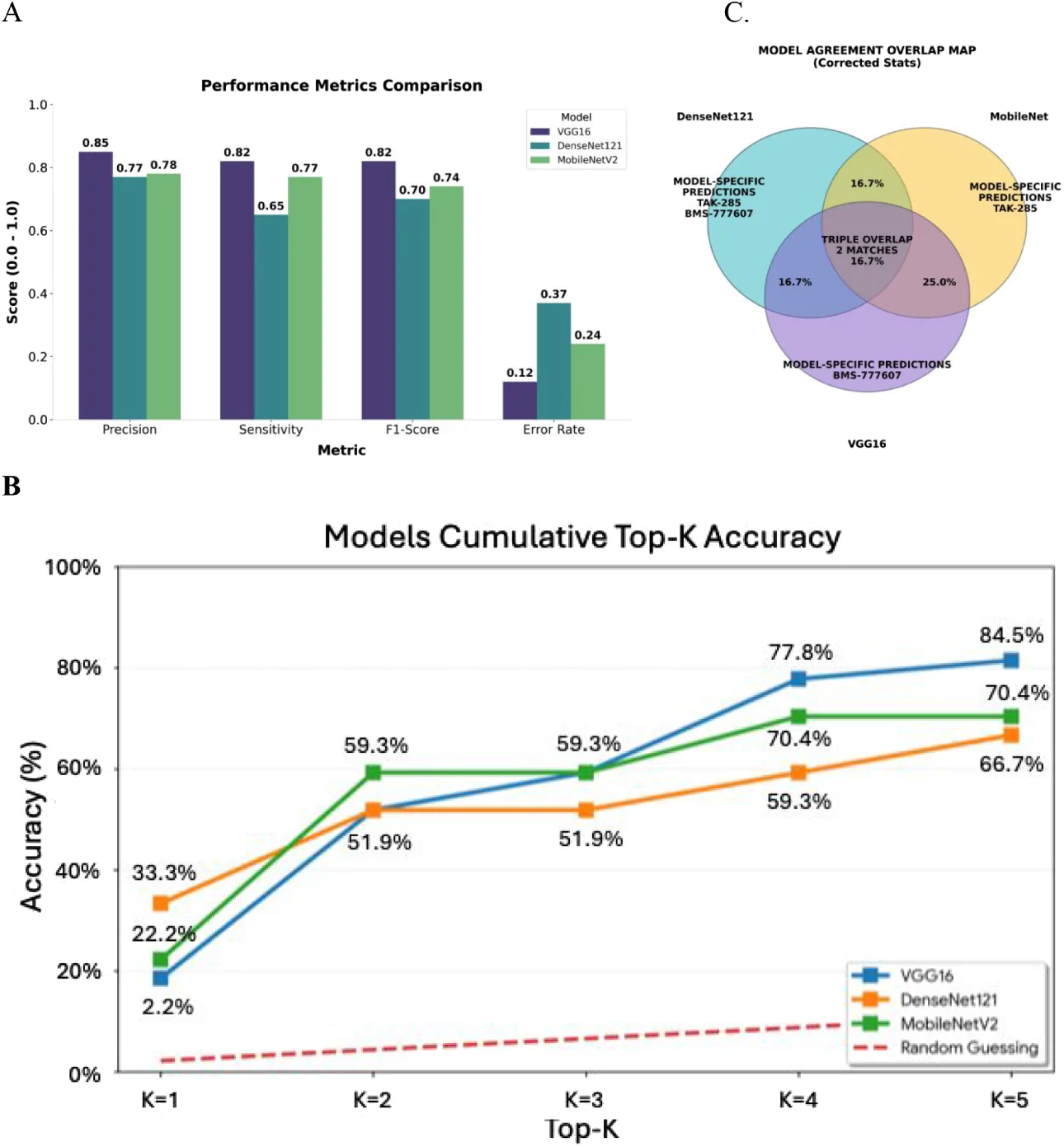
Performance comparison of VGG16, DenseNet121, and MobileNetV2 computer vision models. A) VGG16 outperforms all other models across the four evaluated metrics. The performance difference is statistically significant only between VGG16 and DenseNet121 (*p* < 0.05). B) VGG16 shows lower accuracy for *K* < 3 compared to MobileNetV2 and DenseNet121, but it achieves superior performance for 3 < *K* ≤ 5. All models demonstrate a statistically significant (*p* < 0.05) improvement over a random guess baseline. C) The consistent assignment of common compound-kinase pairs (overlapping region) by all three models suggests the robustness of the QR data.

We further evaluated the sponsor-drug pairing quality of MobileNetV2 and DenseNet121. Their significant overlap (see Figure 7C) suggests our QR data is robust enough to produce consistent patterns in latent space that all three architectures can recognize. For example, VGG16 assigned the same two sponsor compound-kinase pairs (Ponatinib-BRAF and Ponatinib-VGFR) as both DenseNet121 and MobileNet. Again, we turned to using estimated *IC*_50_ values to probe binding interactions. The *IC*_50_ peak frequency (mode) values are 0.2 nM, 13 nM, and 100 nM for VGG16, DenseNet121, and MobileNet, respectively. These results suggest VGG16 identifies a narrower, higher-potency range of sponsor-kinase pairs, whereas the other two models sample broader ranges of binding strength. Furthermore, we calculate the *IC*_50_ shifts produced from this test set to be statistically significant (*p* < 0.05). We therefore determine that other than the processing speed advantage, VGG16 offer consistently differentiable advantage in kinase assignment over both DenseNet121 and MobileNetV2.

### Impact of QR Augmentation on Baseline Performance

Following our systematic testing of the different ML architectures to optimize the VGG16 engine for QRxVision, we shifted our focus on enhancing the input data diversity and improving relevant target proximity in latent space. We initially tried augmenting SMILES strings to generate five different variants of a canonical SMILES with the rationale that increasing the variance in the input data can reciprocate invariance in the output feature. Since each test contains a 5-fold increase in feature data points representation, the computational cost scales equivalently; however the processing time remains practical and usually completes within an average of ≤5 min.

Unexpectedly, the 5-fold data augmentation (A5) slightly worsened QRxVision’s performance, increasing the Top-5 missed rate from ∼29% (*n* = 27) to ∼34% (see Figure 8A). Moreover, we noticed a change in sponsor compound-kinase assignments from the baseline and observed a discrepancy between the hit/missed pairs identified previously for the unaugmented (UA) approach. We hypothesize that the increase in feature vector representations for A5 reached new nearest-neighbor reference vectors, resulting in new sponsor compound-kinase assignments that were either hits or misses. Accordingly, we followed up with a smaller 2-fold SMILES augmentation (A2) to control the off-target data drift in latent space. Despite this adjustment, the resulting performance did not improve the Top-5 missed rate; we even observed a lower mean reciprocal rank than in the A5 approach.

**Figure 8:**
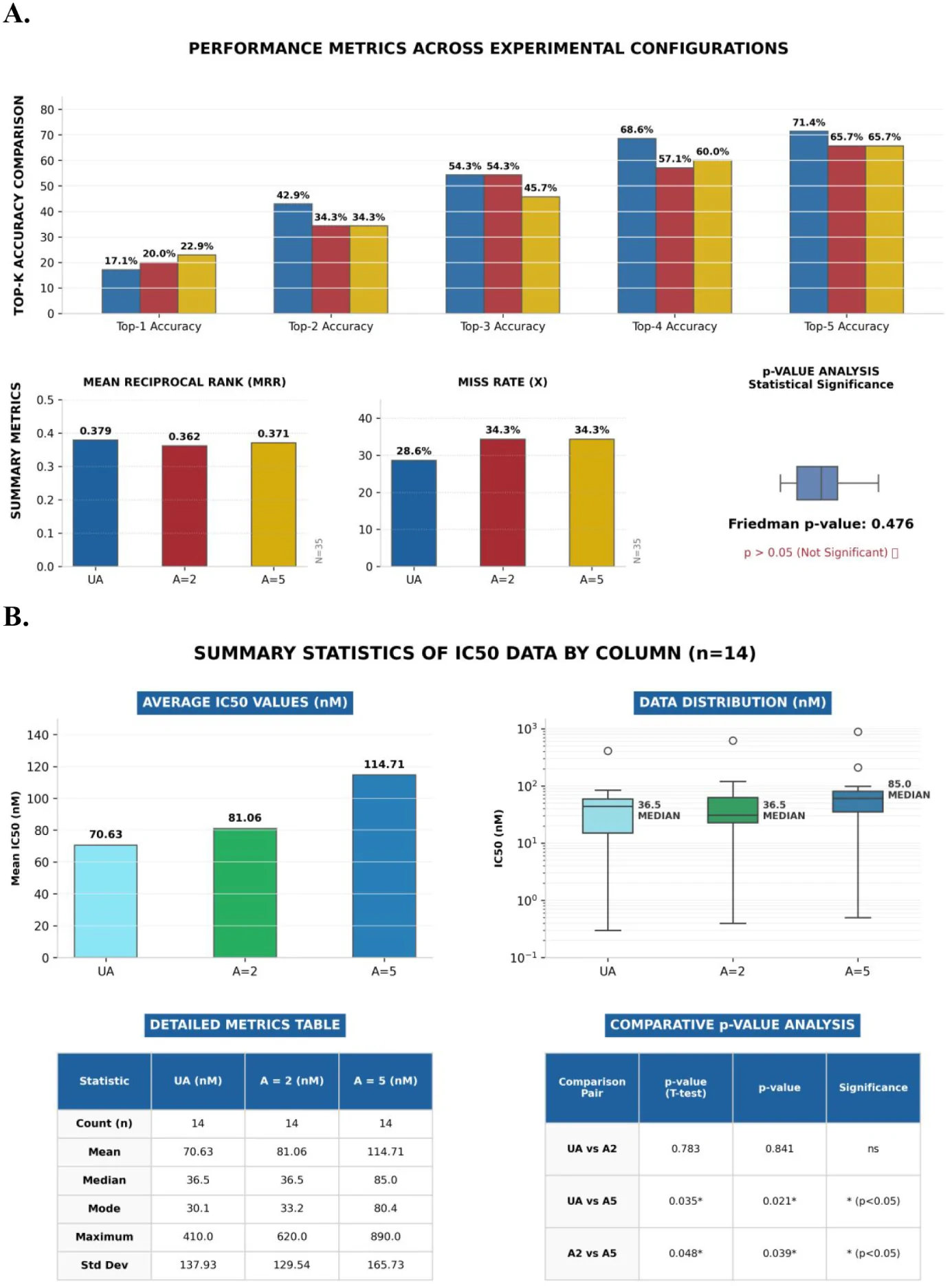
Effect of SMILES string augmentations on kinase assignment performance. UA refers to unaugmented data points. A = 2 and A = 5 represent 2-fold (two SMILES string variants) and 5-fold augmentations, respectively. A) For K > 1, UA demonstrates improved accuracy, a lower missed rate, and a marginally higher MRR; however, these gains are not statistically significant (*p* > 0.05). B) Compound-kinase pairs from UA set exhibit significantly lower average IC50 values, indicating a distribution shifted toward stronger molecular interactions. This increase in potency is statistically significant (*p* < 0.05).

Overall, differences in Top-*k* accuracy, MRR, and missed rates between the three augmentation cohorts were not statistically significant (*p* > 0.05). We therefore cross-referenced databases to estimate *IC*_50_ values for the *n* = 12 test samples that were successfully assigned by all three approaches (UA, A2, and A5). The remaining 13 samples from the *n* = 27 test set were incorrectly assigned by one or more methods and were excluded from further analysis. Figure 8B demonstrates that the UA approach more frequently assigned pairs with lower *IC*_50_ values (mode = 30.1nM) and yielded the lowest overall average (mode = 70.6 nM). Furthermore, *IC*_50_ variance analysis (*p* < 0.05) revealed a statistically significant distinction between the UA and A5 approaches, whereas no such difference was observed between UA and A2.

Our evaluation of 2-fold and 5-fold SMILES augmentations revealed a significant decline in Top-5 target retrieval accuracy. Since a randomized shuffle of a SMILES string fundamentally alters the character sequence, it generates a completely different QR code matrix, causing severe vector drift in the VGG16 latent space. While traditional chemical language models require extensive training augmentation to learn invariance to alternative SMILES strings, QRxVision achieves structural invariance through strict operational constraint. Generating a canonical SMILES string using RDKit is a computationally instantaneous, deterministic, and universally reproducible procedure. By enforcing a strict canonical input convention during the preprocessing stage, alternative structural permutations are eliminated before image rendering. This design framework forces the molecule into a single, unambiguous topological state, ensuring stable target assignments without requiring the deep learning backbone to natively resolve text-shuffling variations.

### Cheminformatics Baseline Benchmark

To evaluate the value added by employing QR image embedding in QRxVision, we evaluated traditional baseline frameworks in parallel using raw, multimodal numerical vectors as input. All three baseline configurations utilized cosine similarity for nearest-neighbor target retrieval calculations. However, for Baselines 1 and 2, the final joint similarity space was derived using an element-wise Hadamard product, whereas the Baseline 3 similarity matrix was calculated directly from a single concatenated vector space. This architectural distinction ensures that Baseline 3 reflects the performance of the QRxVision data input representation prior to pixel conversion, isolating the explicit effects of the QR image embedding VGG16 feature extraction steps. The results in Figure 9 demonstrate that these traditional approaches employing raw descriptor vectors encounter a significant performance ceiling, with Baseline 1 yielding a Top-5 accuracy of 22.6%, while Baselines 2 and 3 both produce an equally poor accuracy of 27.4%. This large performance gap compared to QRxVision verifies that encoding multimodal descriptors into a spatial image leverages deep computer architectures to capture complex, nonlinear dependencies that traditional lower-dimension linear vector space processing fails to resolve.

**Figure 9:**
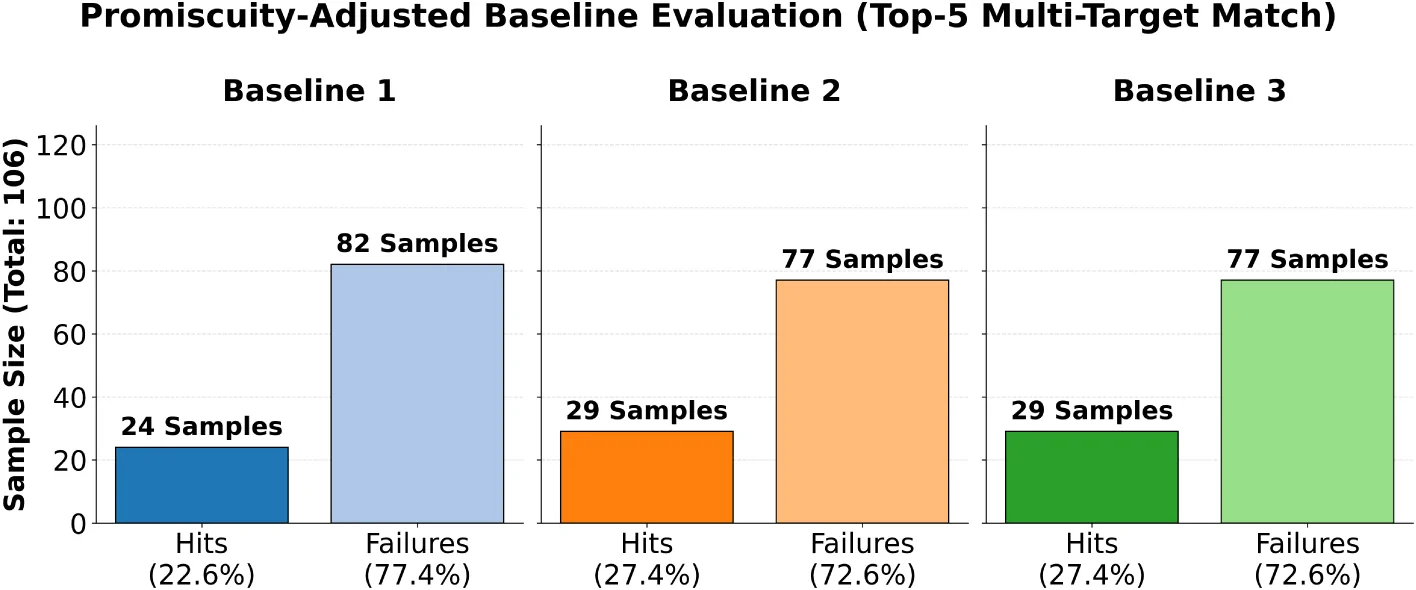
Performances for raw vector multimodal baseline architectures. Baseline 1 utilizes late score fusion on stacked hybrid drug descriptors (Morgan fingerprints + native drug descriptors) multiplied elementwise to target similarity matrices. Baseline 2 applies late-stage score fusion to standard Morgan fingerprints and target arrays. Baseline 3 executes early stage feature fusion by directly concatenating raw tabular drug and target descriptors into a single joint matrix. Top-5 accuracy rates for viable target retrieval (22.6%, 27.4%, and 27.4%, respectively) are significantly lower than the 71% achieved by the QRxVision image-based architecture.

### Deep ML Architecture Benchmark

Validating the true computational value of image vision required comparing QRxVision against high-capacity deep learning models operating directly on raw 1D tabular vector spaces. For this task we implemented a deep multilayer perceptron (MLP) and a multimodal cross-attention network utilizing structural Morgan fingerprints. Both models underwent hyperparameter optimization to support optimal performance. The CMC curve analysis demonstrates a significant (*p* < 0.05) performance separation across all target retrieval ranks (Figure 10). At Rank 1, MLP and Cross-Attention achieve target assignment accuracy rates of 8.6% and 14.3%, respectively. Extending the trial attempt to a Rank 5 threshold yields a final accuracy of 36.4% for the MLP architecture and 40.9% for the Cross-Attention network. In comparison, QRxVision outperforms both models, achieving a Top-5 target capture accuracy of 71.4% on the identical 35-drug validation set.

**Figure 10:**
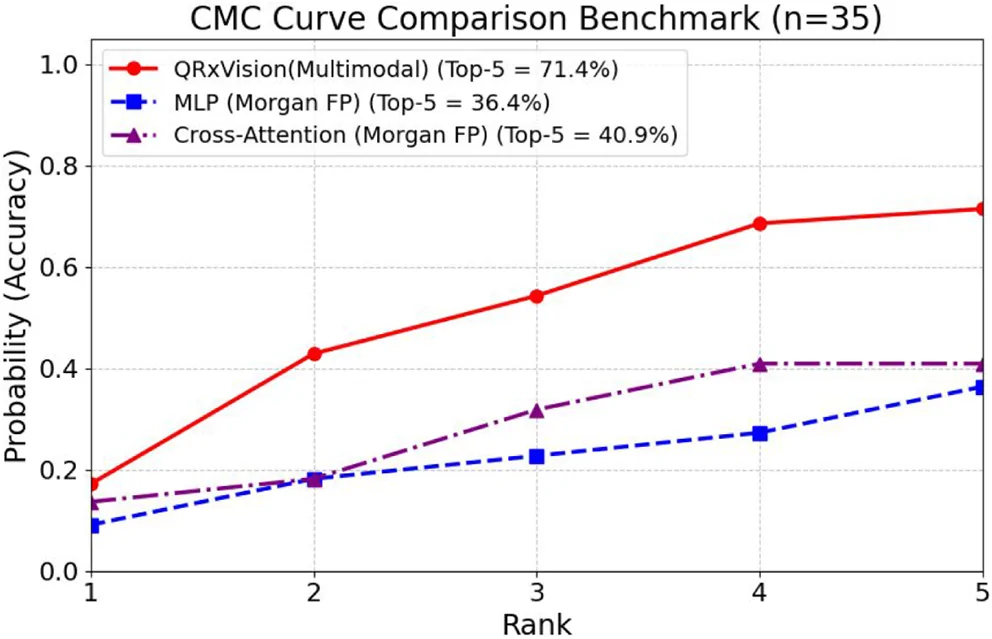
CMC curve comparison against deep baselines. Target retrieval accuracy across Rank 1 to Rank 5. QRxVision significantly outperforms both the optimized 1D multilayer perceptron (*p* = 0.003) and the advanced multimodal Cross-Attention network (*p* = 0.012), supporting the structural advantage of image representation.

Accordingly, these additional results offer good architectural proof regarding the mechanism of our performance gain. The differentiation of QRxVision over deep 1D alternatives indicates that the performance improvement is not simply an artifact of network depth or overall parameter complexity. Instead, we attribute the observed performance gap to leveraging multimodality and image vision for capturing complex structural relationships that deep 1D layers fail to extract.

### Kinase Inhibitor Promiscuity and Model Predictive Power

Figure S1 in Supporting Information illustrates the comprehensive cluster map results for test compound (small circles) kinase assignment via inference-sponsored (large circle) transitive mapping. While 25 viable kinase assignments were confirmed through IC50 validation—affirming the bioactivity of our test set—the raw 71% Top-5 success rate of QRxVision requires contextualization within the framework of kinase promiscuity. This inherent polypharmacology is largely driven by the high structural conservation of the ATP-binding pocket. Specifically, ATP-competitive inhibitors (Type I, I1/2, and Type II) exhibit baseline promiscuity rates of approximately 50%, 50%, and 38%, respectively, whereas non-ATP pocket binders (Type III) show significantly lower multitarget binding rate of ∼8%.

We observed a weighted average promiscuity rate of ∼41% across our 25-compound test set (class = 45), corresponding to a high random success probability for Top-5 assignments (see Figure 11A). Under this broad baseline, the random Top-5 success rate reaches ∼19% (6.55% baseline + 12.45% promiscuous baseline), resulting in a Predictive Power Score (PPS) of ∼0.64 (model Top-5 accuracy = 71%). Although we achieve good PPS in hit identification,^34,35^ when we consider drug-target interaction this current metric setup does not give quantitative information about the quality of the hits. To elucidate this, we redefined the predictive baseline by implementing a high-potency threshold (*pIC*_50_ ≥ 7.5) based on the benchmarks established by Davis et al.^36^ Under this rigorous constraint, QRxVision achieves a PPS of 0.42 and a ∼7× enrichment factor (see Figure 11B). Crucially, the best hits identified by the model achieved an average potency (*pIC*_50_ = 8.3) (for a complete list see Table S3 in Supporting Information), representing a 630-fold potency gain over the background noise floor (*pIC*_50_ = 5.5–3,162 nM). This shift confirms that QRxVision captures specific high-affinity structural motifs via feature map rather than merely sampling the promiscuous regions of latent space.

**Figure 11:**
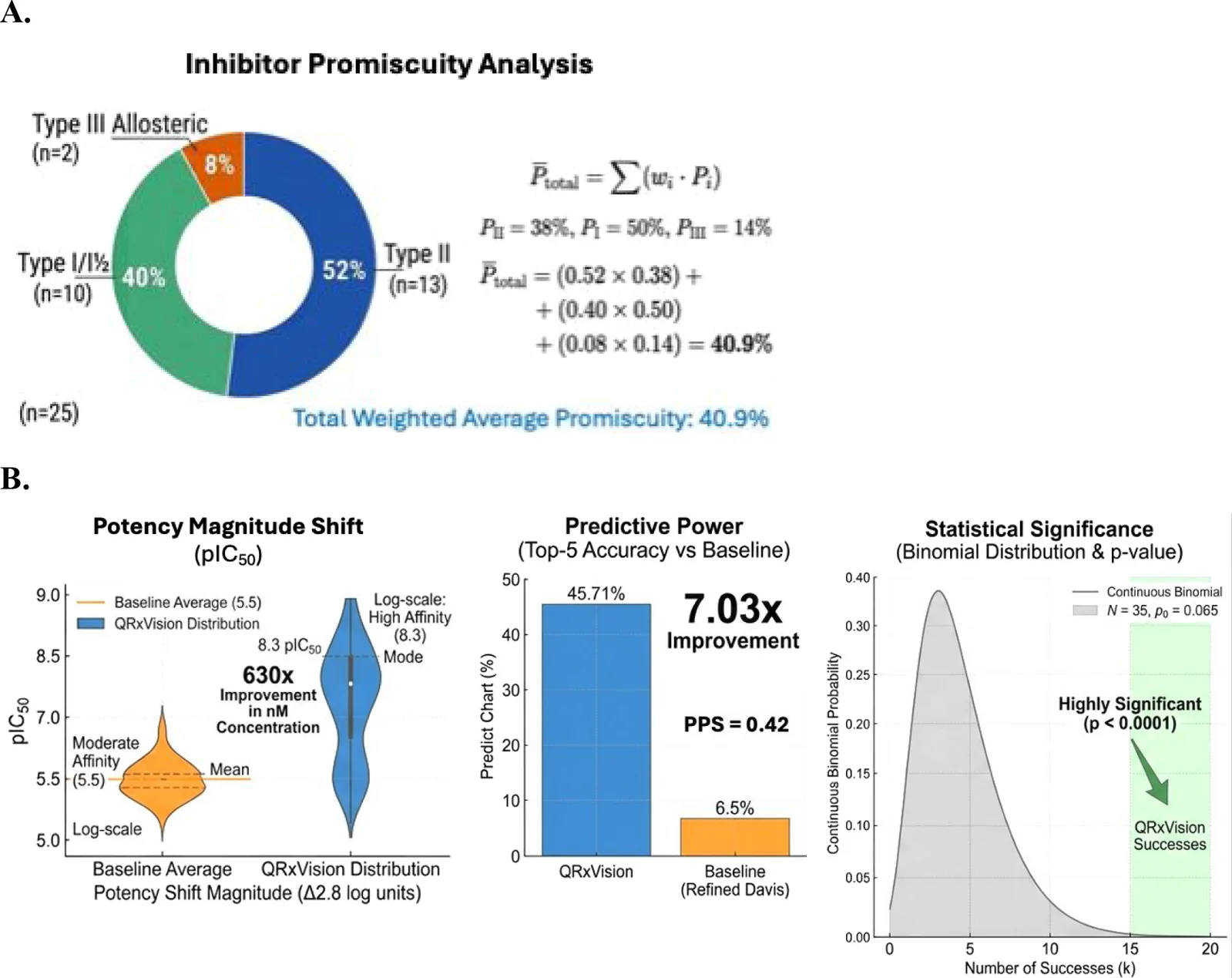
Adjusting performance metrics to account for kinase inhibitor promiscuity. A) Determination of the average promiscuity rate for test samples using a population-weighted average and inhibitor-type-specific rates. Approximately 40.9% of compounds in the test set bind to more than one kinase. B) Increasing the screening threshold from general binding to high-potency (*pIC*_50_ ≥ 7.5) represents a 630-fold improvement in *IC*_50_ concentration, leading to a 7-fold enrichment in predictive precision over Davis baseline. The PPS was calculated using the Davis baseline.

## Conclusions

In this work, we established QRxVision, a domain-specific self-supervised model that achieves value innovation by bypassing high-compute molecular simulations or data intensive models in favor of efficient, QR code images. By QR-embedding multimodality-associated parameters (MAP) data into a high-depth latent space via the VGG16 CNN architecture, we transformed a complex chemical-biological problem into a streamlined image-recognition task. Our analysis of feature vectors using cosine similarity successfully identified viable nearest neighbors, enabling accurate kinase assignment through sponsor compound lead transitive relationships. This approach achieved an overall 71% Top-5 accuracy for compound-kinase assignments.

While QRxVision currently lacks the sensitivity to capture complex multi-pharmacological vectors in the promiscuous kinase domain, it offers significant utility for high-potency screening. By focusing on pairs with a *pIC*_50_ ≥ 7.5, we observed a substantial boost in predictive power (0.42) and accuracy enrichment (7-fold) over the Davis baseline. This method creates a new approach for drug screening by providing a low-cost, high-speed alternative to traditional unimodal-based models or traditional docking studies. Future integration of larger reference compound data will further refine relevant latent space mapping, increasing both general accuracy and the precision of high-potency target identification.

## Supplementary Material



## Data Availability

QRxVision data, documentation, and executable model are accessible at https://github.com/mitkeng/QRxVision

## Notes

Validated reference compounds, test compound target assignment results, predicted test set pIC50 results (PDF)

## References

[1] Simonyan, K. ; Zisserman, A. Very Deep Convolutional Networks for Large-Scale Image Recognition arXiv 2015 10.48550/arXiv.1409.1556

[2] Science, I. I. F. H. D. Global Oncology Trends 2025: Adopting New Therapies as Modalities Shift and Expenditures Rise; IQVIA Institute for Human Data Science, 2025. https://www.iqvia.com/insights/the-iqvia-institute/reports-and-publications/reports/global-oncology-trends-2025.

[3] Siegel R. L., Giaquinto A. N., Jemal A. (2024). Cancer statistics, 2024. Cancer J. Clin..

[4] Baldi, S. ; Long, N. ; Ma, S. ; Liu, L. ; Al-Danakh, A. ; Yang, Q. ; Deng, X. ; Xie, J. ; Tang, H. Advancements in Protein Kinase Inhibitors: From Discovery to Clinical Applications. Research, 8, 0747. 10.34133/research.0747 PMC12180671 40547482

[5] Jain V., Bage S., Dhiman N., Singh S., Yadav A., Brünnert D., Sawant D. M., Goyal P. (2026). Relevance
of EGFR-HER2
Dual Inhibition in Breast Cancer. Targets.

[6] Murphy C. G., Modi S. (2009). HER2 breast cancer
therapies: a review. Biologics:
targets Therapy.

[7] Bray F., Laversanne M., Sung H., Ferlay J., Siegel R. L., Soerjomataram I., Jemal A. (2024). Global cancer statistics 2022: GLOBOCAN
estimates of incidence and mortality worldwide for 36 cancers in 185
countries. Cancer J. Clin..

[8] Ocana A., Pandiella A., Privat C., Bravo I., Luengo-Oroz M., Amir E., Gyorffy B. (2025). Integrating artificial intelligence
in drug discovery and early drug development: a transformative approach. Biomarker Res..

[9] Niazi S. K., Mariam Z. (2024). Computer-Aided Drug Design and Drug
Discovery: A Prospective
Analysis. Pharmaceuticals.

[10] Blanco-González A., Cabezón A., Seco-González A., Conde-Torres D., Antelo-Riveiro P., Piñeiro Á., Garcia-Fandino R. (2023). The Role of
AI in Drug Discovery: Challenges, Opportunities, and Strategies. Pharmaceuticals.

[11] Bobolea S.-S., Hinoveanu M.-I., Dimitriu A., Braşoveanu M.-A., Iliescu C.-N., Dinu-Pîrvu C.-E., Ghica M. V., Anuţa V., Popa L., Prisada R. M. (2025). Exploring
Artificial Intelligence’s
Potential to Enhance Conventional Anticancer Drug Development. Drug Dev. Res..

[12] Selvaraj C., Chandra I., Singh S. K. (2022). Artificial
intelligence and machine
learning approaches for drug design: challenges and opportunities
for the pharmaceutical industries. Mol. Diversity.

[13] Ferreira F. J. N., Carneiro A. S. (2025). AI-Driven Drug Discovery: A Comprehensive Review. ACS Omega.

[14] Serrano D. R., Luciano F. C., Anaya B. J., Ongoren B., Kara A., Molina G., Ramirez B. I., Sánchez-Guirales S. A., Simon J. A., Tomietto G., Rapti C., Ruiz H. K., Rawat S., Kumar D., Lalatsa A. (2024). Artificial Intelligence
(AI). Applications In Drug Discovery And Drug Delivery: revolutionizing
Personalized Medicine. Pharmaceutics.

[15] Singh R., Barsainyan A. A., Irfan R., Amorin C. J., He S., Davis T., Thiagarajan A., Sankaran S., Chithrananda S., Ahmad W., Jones D., McLoughlin K., Kim H., Bhutani A., Sathyanarayana S. V., Viswanathan V., Allen J. E., Ramsundar B. (2026). ChemBERTa-3: an open source training
framework for chemical foundation models. Digital
Discovery.

[16] Goh G. B., Hodas N. O., Siegel C., Vishnu A. (2017). SMILES2Vec: An Interpretable
General-Purpose Deep Neural Network for Predicting Chemical Properties. arXiv.

[17] Luo, Y. ; Liu, X. Y. ; Yang, K. ; Huang, K. ; Hong, M. ; Zhang, J. ; Wu, Y. ; Nie, Z. Toward Unified AI Drug Discovery with Multimodal Knowledge. Health Data Sci., 4, 0113. 10.34133/hds.0113 PMC10886071 38486623

[18] Goh G. B., Siegel C., Vishnu A., Hodas N. O., Baker N. (2017). Chemception:
A Deep Neural Network with Minimal Chemistry Knowledge Matches the
Performance of Expert-developed QSAR/QSPR Models. arXiv.

[19] Gómez-Bombarelli R., Wei J. N., Duvenaud D., Hernández-Lobato J. M., Sánchez-Lengeling B., Sheberla D., Aguilera-Iparraguirre J., Hirzel T. D., Adams R. P., Aspuru-Guzik A. (2018). Automatic
Chemical Design Using a Data-Driven Continuous Representation of Molecules. ACS Cent. Sci..

[20] Zeng X., Xiang H., Yu L., Wang J., Li K., Nussinov R., Cheng F. (2022). Accurate prediction
of molecular
properties and drug targets using a self-supervised image representation
learning framework. Nat. Mach. Intell.

[21] Uthayakumar H., Rahul K. K., Jain R., Kumar R., Patra T. K. (2024). QRChEM:
A Deep Learning Framework for Materials Property Prediction and Design
Using QR Codes. ACS Eng. Au.

[22] Zhou Y., Zhang Y., Zhao D., Yu X., Shen X., Zhou Y., Wang S., Qiu Y., Chen Y., Zhu F. (2024). TTD: Therapeutic Target Database
describing target druggability information. Nucleic Acids Res..

[23] Krapp L. F., Abriata L. A., Cortés
Rodriguez F., Dal Peraro M. (2023). PeSTo: parameter-free
geometric deep learning for accurate prediction of protein binding
interfaces. Nat. Commun..

[24] Brielle E. S., Arkin I. T. (2020). Quantitative Analysis
of Multiplex H-Bonds. J. Am. Chem. Soc..

[25] Addisu E. G., Yirga T. G., Yirga H. G., Yehuala A. D. (2025). Transfer learning-based
hybrid VGG16-machine learning approach for heart disease detection
with explainable artificial intelligence. Front.
Artif. Intell..

[26] Ibrahim A. A. M. S., Tapamo J. R. (2024). Transfer learning-based
approach using new convolutional
neural network classifier for steel surface defects classification. Sci. Afr..

[27] Obaido G., Mienye I. D., Egbelowo O. F., Emmanuel I. D., Ogunleye A., Ogbuokiri B., Mienye P., Aruleba K. (2024). Supervised
machine
learning in drug discovery and development: Algorithms, applications,
challenges, and prospects. Mach. Learn. With
Appl..

[28] Qiao J., Jin J., Wang D., Teng S., Zhang J., Yang X., Liu Y., Wang Y., Cui L., Zou Q., Su R., Wei L. (2025). A self-conformation-aware pre-training framework for molecular property
prediction with substructure interpretability. Nat. Commun..

[29] Wu J., Chen Y., Wu J., Zhao D., Huang J., Lin M., Wang L. (2024). Large-scale
comparison of machine learning methods
for profiling prediction of kinase inhibitors. J. Cheminf..

[30] Kuthuru S., Szafran A. T., Stossi F., Mancini M. A., Rao A. (2019). Leveraging
Image-Derived Phenotypic Measurements for Drug-Target Interaction
Predictions. Cancer Informatics.

[31] Karaman M. W., Herrgard S., Treiber D. K., Gallant P., Atteridge C. E., Campbell B. T., Chan K. W., Ciceri P., Davis M. I., Edeen P. T., Faraoni R., Floyd M., Hunt J. P., Lockhart D. J., Milanov Z. V., Morrison M. J., Pallares G., Patel H. K., Pritchard S., Wodicka L. M., Zarrinkar P. P. (2008). A quantitative
analysis of kinase inhibitor selectivity. Nat.
Biotechnol..

[32] Burt T., Button K. S., Thom H. H. Z., Noveck R. J., Munafò M. R. (2017). The Burden
of the “False-Negatives” in Clinical Development: Analyses
of Current and Alternative Scenarios and Corrective Measures. Clin. Transl. Sci..

[33] Hicks S. A., Strümke I., Thambawita V., Hammou M., Riegler M. A., Halvorsen P., Parasa S. (2022). On evaluation metrics for medical
applications of artificial intelligence. Sci.
Rep..

[34] Vu-Han T.-L., Schettino R. B., Weiß C., Perka C., Winkler T., Sunkara V., Pumberger M. (2024). An interpretable data-driven prediction
model to anticipate scoliosis in spinal muscular atrophy in the era
of (gene-). therapies. Sci. Rep..

[35] Gribova V., Navalikhina A., Lysenko O., Calligaro C., Lebaudy E., Deiber L., Senger B., Lavalle P., Vrana N. E. (2021). Prediction of coating
thickness for polyelectrolyte
multilayers via machine learning. Sci. Rep..

[36] Davis M. I., Hunt J. P., Herrgard S., Ciceri P., Wodicka L. M., Pallares G., Hocker M., Treiber D. K., Zarrinkar P. P. (2011). Comprehensive
analysis of kinase inhibitor selectivity. Nat.
Biotechnol..

