# Supplementary material for: QRxVision: Multimodal QR Encoding and Computer Vision for Domain-Specific Drug Discovery
Source: J Chem Inf Model. 2026 Oct 28;66(21):13783–94. doi: 10.1021/acs.jcim.6c01321 (PMC13753511; doi:10.1021/acs.jcim.6c01321)
Supplement: Supplementary file 1 [file ci6c01321_si_001.pdf]

QRxVision: Multimodal QR Encoding and Computer Vision for Domain-Specific Drug Discovery

Mithony Keng and Kenneth M Merz, Jr.*

Department of Chemistry, Michigan State University,

East Lansing, Michigan 48824, United States

Department of Biochemistry and Molecular Biology, Michigan State University,

East Lansing, Michigan 48824, United States

*Corresponding Author: Kenneth M. Merz

*Corresponding Author Email: merz@chemistry.msu.edu

Table S1. 111 reference compounds used in transitive matching for drug-kinase target assignment

Ref Compound	SMILES
Pemigatinib	CCN1C2=C3C=C(NC3=NC=C2CN(C1=O)C4=C(C(=CC(=C4F)OC)OC)F)CN5CCOCC5
Erdafitinib	CC(C)NCCN(C1=CC2=NC(=CN=C2C=C1)C3=CN(N=C3)C)C4=CC(=CC(=C4)OC)OC
Lapatinib	CS(=O)(=O)CCNCC1=CC=C(O1)C2=CC3=C(C=C2)N=CN=C3NC4=CC(=C(C=C4)OCC5=CC(=CC=C5)F)Cl
Brigatinib	CN1CCN(CC1)C2CCN(CC2)C3=CC(=C(C=C3)NC4=NC=C(C(=N4)NC5=CC=CC=C5P(=O)(C)C)Cl)OC
Actinonin	CCCCC[C@H](CC(=O)NO)C(=O)N[C@@H](C(C)C)C(=O)N1CCC[C@H]1CO
Tofacitinib	C[C@@H]1CCN(C[C@@H]1N(C)C2=NC=NC3=C2C=CN3)C(=O)CC#N
Baricitinib	CCS(=O)(=O)N1CC(C1)(CC#N)N2C=C(C=N2)C3=C4C=CNC4=NC=N3
Lapatinib	CS(=O)(=O)CCNCC1=CC=C(O1)C2=CC3=C(C=C2)N=CN=C3NC4=CC(=C(C=C4)OCC5=CC(=CC=C5)F)Cl
L-betagamma-meATP	C1=NC(=C2C(=N1)N(C=N2)[C@H]3[C@@H]([C@@H]([C@H](O3)COP(=O)(O)OP(=O)(CP(=O)(O)O)O)O)O)N
Gilteritinib	CCC1=C(N=C(C(=N1)C(=O)N)NC2=CC(=C(C=C2)N3CCC(CC3)N4CCN(CC4)C)OC)NC5CCOCC5
Myo-inositol hexaphosphate	C1(C(C(C(C(C1OP(=O)(O)O)OP(=O)(O)O)OP(=O)(O)O)OP(=O)(O)O)OP(=O)(O)O)OP(=O)(O)O
Imatinib	CC1=C(C=C(C=C1)NC(=O)C2=CC=C(C=C2)CN3CCN(CC3)C)NC4=NC=CC(=N4)C5=CN=CC=C5
MMI270	CC(C)C(C(=O)NO)N(CC1=CN=CC=C1)S(=O)(=O)C2=CC=C(C=C2)OC
Imatinib	CC1=C(C=C(C=C1)NC(=O)C2=CC=C(C=C2)CN3CCN(CC3)C)NC4=NC=CC(=N4)C5=CN=CC=C5

Adenosine	C1=NC(=C2C(=N1)N(C=N2)[C@H]3[C@@H]([C@@H]([C@H](O3)CO)O)O)N
Ponatinib	CC1=C(C=C(C=C1)C(=O)NC2=CC(=C(C=C2)CN3CCN(CC3)C)C(F)(F)F)C#CC4=CN=C5N4N=CC=C5
Ponatinib	CC1=C(C=C(C=C1)C(=O)NC2=CC(=C(C=C2)CN3CCN(CC3)C)C(F)(F)F)C#CC4=CN=C5N4N=CC=C5
Sirolimus	C[C@@H]1CC[C@H]2C[C@@H](/C(=C/C=C/C=C/[C@H](C[C@H](C(=O)[C@@H]([C@@H](/C(=C/[C@H](C(=O)C[C@H](OC(=O)[C@@H]3CCCCN3C(=O)C(=O)[C@@]1(O2)O)[C@H](C)C[C@@H]4CC[C@H]([C@@H](C4)OC)O)C)/C)O)OC)C)C)/C)OC
Entrectinib	CN1CCN(CC1)C2=CC(=C(C=C2)C(=O)NC3=NNC4=C3C=C(C=C4)CC5=CC(=CC(=C5)F)F)NC6CCOCC6
Dasatinib	CC1=C(C(=CC=C1)Cl)NC(=O)C2=CN=C(S2)NC3=CC(=NC(=N3)C)N4CCN(CC4)CCO
Ibrutinib	C=CC(=O)N1CCC[C@H](C1)N2C3=NC=NC(=C3C(=N2)C4=CC=C(C=C4)OC5=CC=CC=C5)N
Sunitinib	CCN(CC)CCNC(=O)C1=C(NC(=C1C)/C=C\2/C3=C(C=CC(=C3)F)NC2=O)C
Pexidartinib	C1=CC(=NC=C1CC2=CNC3=C2C=C(C=N3)Cl)NCC4=CN=C(C=C4)C(F)(F)F
Tivozanib	CC1=CC(=NO1)NC(=O)NC2=C(C=C(C=C2)OC3=C4C=C(C(=CC4=NC=C3)OC)OC)Cl
Sunitinib	CCN(CC)CCNC(=O)C1=C(NC(=C1C)/C=C\2/C3=C(C=CC(=C3)F)NC2=O)C
Ceritinib	CC1=CC(=C(C=C1C2CCNCC2)OC(C)C)NC3=NC=C(C(=N3)NC4=CC=CC=C4S(=O)(=O)C(C)C)Cl
Alectinib	CCC1=CC2=C(C=C1N3CCC(CC3)N4CCOCC4)C(C5=C(C2=O)C6=C(N5)C=C(C=C6)C#N)(C)C
Liothyronine	C1=CC(=C(C=C1OC2=C(C=C(C=C2I)CC(C(=O)O)N)I)I)O
Testosterone	CC12CCC3C(C1CCC2O)CCC4=CC(=O)CCC34C
Testosterone	CC12CCC3C(C1CCC2O)CCC4=CC(=O)CCC34C
Exemestane	C[C@]12CC[C@H]3[C@H]([C@@H]1CCC2=O)CC(=C)C4=CC(=O)C=C[C@]34C
Phosphonotryosine	C1=CC(=CC=C1C[C@@H](C(=O)O)N)OP(=O)(O)O
isopropyl dihydrogen phosphate	CC(C)OP(=O)(O)O
Benzamidine	C1=CC=C(C=C1)C(=N)N
Abemaciclib	CCN1CCN(CC1)CC2=CN=C(C=C2)NC3=NC=C(C(=N3)C4=CC5=C(C(=C4)F)N=C(N5C(C)C)C)F
Abemaciclib	CCN1CCN(CC1)CC2=CN=C(C=C2)NC3=NC=C(C(=N3)C4=CC5=C(C(=C4)F)N=C(N5C(C)C)C)F
Abemaciclib	CCN1CCN(CC1)CC2=CN=C(C=C2)NC3=NC=C(C(=N3)C4=CC5=C(C(=C4)F)N=C(N5C(C)C)C)F
Palbociclib	CC1=C(C(=O)N(C2=NC(=NC=C12)NC3=NC=C(C=C3)N4CCNCC4)C5CCCC5)C(=O)C
Bimatoprost	CCNC(=O)CCCC=CCC1C(CC(C1C=CC(CCC2=CC=CC=C2)O)O)O
Gliclazide	CC1=CC=C(C=C1)S(=O)(=O)NC(=O)NN2CC3CCCC3C2
Phosphonotryosine	C1=CC(=CC=C1CC(C(=O)O)N)OP(=O)(O)O
Staurosporine	CC12C(C(CC(O1)N3C4=CC=CC=C4C5=C6C(=C7C8=CC=CC=C8N2C7=C53)CNC6=O)NC)OC
L-aspartic acid	C([C@@H](C(=O)O)N)C(=O)O
L-methionine	CSCC[C@@H](C(=O)O)N
Taxol	CC1=C2C(C(=O)C3(C(CC4C(C3C(C(C2(C)C)(CC1OC(=O)C(C(C5=CC=CC=C5)NC(=O)C6=CC=CC=C6)O)O)OC(=O)C7=CC=CC=C7)(CO4)OC(=O)C)O)C)OC(=O)C
Vincristine	CCC1(CC2CC(C3=C(CCN(C2)C1)C4=CC=CC=C4N3)(C5=C(C=C6C(=C5)C78CCN9C7C(C=CC9)(C(C(C8N6C=O)(C(=O)OC)O)OC(=O)C)CC)OC)C(=O)OC)O

3-[2-[6-(4-aminophenyl)imidazo[1,2-a]pyrazin-3-yl]ethynyl]-2-methyl-N-[3-(4-methylpiperazin-1-yl)-5-propan-2-ylphenyl]benzamide	CC1=C(C=CC=C1C(=O)NC2=CC(=CC(=C2)C(C)C)N3CCN(CC3)C)C#CC4=CN=C5N4C=C(N=C5)C6=CC=C(C=C6)N
Alpelisib	CC1=C(SC(=N1)NC(=O)N2CCCC2C(=O)N)C3=CC(=NC=C3)C(C)(C)C(F)(F)F
Taselisib	CC1=NN(C(=N1)C2=CN3CCOC4=C(C3=N2)C=CC(=C4)C5=CN(N=C5)C(C)(C)C(=O)N)C(C)C
PI3K-IN-9	CC1CC1C(=O)N2CCN(CC2C)C3=CN=C(C(=N3)C4=CN(N=C4)C)C#N
Copanlisib	COC1=C(C=CC2=C3NCCN3C(=NC(=O)C4=CN=C(N=C4)N)N=C21)OCCCN5CCOCC5
Buparlisib	C1COCCN1C2=NC(=NC(=C2)C3=CN=C(C=C3C(F)(F)F)N)N4CCOCC4
Rucaparib	CNCC1=CC=C(C=C1)C2=C3CCNC(=O)C4=C3C(=CC(=C4)F)N2
Olaparib	C1CC1C(=O)N2CCN(CC2)C(=O)C3=C(C=CC(=C3)CC4=NNC(=O)C5=CC=CC=C54)F
ATP	C1=NC(=C2C(=N1)N(C=N2)C3C(C(C(O3)COP(=O)(O)OP(=O)(O)OP(=O)(O)O)O)O)N
Crizotinib	CC(C1=C(C=CC(=C1Cl)F)Cl)OC2=C(N=CC(=C2)C3=CN(N=C3)C4CCNCC4)N
Crizotinib	CC(C1=C(C=CC(=C1Cl)F)Cl)OC2=C(N=CC(=C2)C3=CN(N=C3)C4CCNCC4)N
PF-06463922	CC1C2=C(C=CC(=C2)F)C(=O)N(CC3=NN(C(=C3C4=CC(=C(N=C4)N)O1)C#N)C)C
24(S), 25-epoxycholesterol	CC(CCC1C(O1)(C)C)C2CCC3C2(CCC4C3CC=C5C4(CCC(C5)O)C)C
Cholesterol	CC(C)CCCC(C)C1CCC2C1(CCC3C2CC=C4C3(CCC(C4)O)C)C
Ozanimod	CC(C)OC1=C(C=C(C=C1)C2=NC(=NO2)C3=C4CCC(C4=CC=C3)NCCO)C#N
Cenerimod	CCC1=C(C(=CC(=C1)C2=NOC(=N2)C3=CC(=NC(=C3)OC)C4CCCC4)C)OCC(CO)O
Cholic acid	CC(CCC(=O)O)C1CCC2C1(C(CC3C2C(CC4C3(CCC(C4)O)C)O)O)C
Abiraterone	CC12CCC(CC1=CCC3C2CCC4(C3CC=C4C5=CN=CC=C5)C)O
Pregnenolone	CC(=O)C1CCC2C1(CCC3C2CC=C4C3(CCC(C4)O)C)C
2'-deoxythymidine triphosphate	CC1=CN(C(=O)NC1=O)C2CC(C(O2)COP(=O)(O)OP(=O)(O)OP(=O)(O)O)O
P(1)-(Adenosine-5')-P(5)-(thymidine-5')-pentaphosphate	CC1=CN(C(=O)NC1=O)C2CC(C(O2)COP(=O)(O)OP(=O)(O)OP(=O)(O)OP(=O)(O)OCC3C(C(C(O3)N4C=NC5=C(N=CN=C54)N)O)O)O
Y35	[B-]1(OC2=C(O1)C=C(C=C2)C(=O)N3CCC(CC3)C4=CC=CC(=C4)CN)(C5=CC(=CC=C5)C6=CC(=CC=C6)C(=O)N7CCC(CC7)C8=CC=CC(=C8)CN)O
Imatinib	CC1=C(C=C(C=C1)NC(=O)C2=CC=C(C=C2)CN3CCN(CC3)C)NC4=NC=CC(=N4)C5=CN=CC=C5
GS-9973	C1COCCN1C2=CC=C(C=C2)NC3=NC(=CN4C3=NC=C4)C5=CC6=C(C=C5)C=NN6
Tranexamic Acid	C1CC(CCC1CN)C(=O)O
Raloxifene	C1CCN(CC1)CCOC2=CC=C(C=C2)C(=O)C3=C(SC4=C3C=CC(=C4)O)C5=CC=C(C=C5)O

Flavin-Adenine Dinucelotide	CC1=CC2=C(C=C1C)N(C3=NC(=O)NC(=O)C3=N2)CC(C(C(COP(=O)(O)OP(=O)(O)OCC4C(C(C(O4)N5C=NC6=C(N=CN=C65)N)O)O)O)O)O
Sunitinib	CCN(CC)CCNC(=O)C1=C(NC(=C1C)C=C2C3=C(C=C(=C3)F)NC2=O)C
L10BFL	CC1C2=C(C=CC(=C2Cl)F)C(=O)N(CC3=C(C(=NN3CCO)C)C4=CN=C(C(=N4)O1)N)C
TAK-285	CC(C)(CC(=O)NCCN1C=CC2=C1C(=NC=N2)NC3=CC(=C(C=C3)OC4=CC=CC(=C4)C(F)(F)F)Cl)O
AZD-1480	C[C@H](NC1=NC=C(Cl)C(NC2=NNC(C)=C2)=N1)C1=NC=C(F)C=N1
Crizotinib	CC(C1=C(C=CC(=C1Cl)F)Cl)OC2=C(N=CC(=C2)C3=CN(N=C3)C4CCNCC4)N
PF-03715455	CC(C)(C)C1=NN(C(NC(=O)NCC2=CC=CC=C2SC2=CN3C(C=C2)=NN=C3C2=CC=CC=C2SCCO)=C1)C1=CC=C(O)C(Cl)=C1
Gefitinib	COC1=C(OCCCN2CCOCC2)C=C2C(NC3=CC(Cl)=C(F)C=C3)=NC=NC2=C1
Vandetanib	COC1=C(OCC2CCN(C)CC2)C=C2N=CN=C(NC3=C(F)C=C(Br)C=C3)C2=C1
Purvalanol	[H][C@@](CO)(NC1=NC2=C(N=CN2C(C)C)C(NC2=CC=C(C(O)=O)C(Cl)=C2)=N1)C(C)C
MLN8054	OC(=O)C1=CC=C(NC2=NC=C3CN=C(C4=C(C=CC(Cl)=C4)C3=N2)C2=C(F)C=CC=C2F)C=C1
AZD-7762	NC(=O)NC1=C(SC(=C1)C1=CC=CC(F)=C1)C(=O)N[C@H]1CCCNC1
Dasatinib	CC1=NC(NC2=NC=C(S2)C(=O)NC2=C(C)C=CC=C2Cl)=CC(=N1)N1CCN(CCO)CC1
Alvocidib	CN1CC[C@@H]([C@H](O)C1)C1=C(O)C=C(O)C2=C1OC(=CC2=O)C1=CC=CC=C1Cl
Nintedanib	COC(=O)C1=CC=C2C(NC(=O)\C2=C(/NC2=CC=C(C=C2)N(C)C(=O)CN2CCN(C)CC2)C2=CC=CC=C2)=C1
Nilotinib	CC1=CN(C=N1)C1=CC(NC(=O)C2=CC=C(C)C(NC3=NC=CC(=N3)C3=CN=CC=C3)=C2)=CC(=C1)C(F)(F)F
Seliciclib	CC[C@H](CO)NC1=NC(NCC2=CC=CC=C2)=C2N=CN(C(C)C)C2=N1
Motesanib	CC1(C)CNC2=CC(NC(=O)C3=C(NCC4=CC=NC=C4)N=CC=C3)=CC=C12
SB220025	NC1=NC=CC(=N1)C1=C(N=CN1C1CCNCC1)C1=CC=C(F)C=C1
BMS-777607	CCOC1=C(C(=O)NC2=CC=C(OC3=C(Cl)C(N)=NC=C3)C(F)=C2)C(=O)N(C=C1)C1=CC=C(F)C=C1
Neflamapimod	FC1=CC(F)=C(SC2=NN3C=NC(=O)C(=C3C=C2)C2=C(Cl)C=CC=C2Cl)C=C1
Imatinib	CN1CCN(CC2=CC=C(C=C2)C(=O)NC2=CC(NC3=NC=CC(=N3)C3=CN=CC=C3)=C(C)C=C2)CC1
SD-0006	OCC(=O)N1CCC(CC1)C1=NNC(=C1C1=CC=NC=N1)C1=CC=C(Cl)C=C1
PH-797804	CNC(=O)C1=CC=C(C)C(=C1)N1C(C)=CC(OCC2=C(F)C=C(F)C=C2)=C(Br)C1=O
Talmapimod	C[C@@H]1CN([C@H](CN1C(=O)C2=C(C=C3C(=C2)C(=CN3C)C(=O)C(=O)N(C)C)Cl)C)CC4=CC=C(C=C4)F
Sotrastaurin	CN1CCN(CC1)C1=NC(C2=C(C(=O)NC2=O)C2=CNC3=C2C=CC=C3)=C2C=CC=CC2=N1
Balanol	OC(=O)C1=CC=CC(O)=C1C(=O)C1=C(O)C=C(C=C1O)C(=O)O[C@@H]1CCCNC[C@@H]1NC(=O)C1=CC=C(O)C=C1
Tofacitinib	C[C@@H]1CCN(C[C@@H]1N(C)C1=NC=NC2=C1C=CN2)C(=O)CC#N
Dichlororibofuranosylbenzimidazole	OC[C@H]1O[C@H]([C@H](O)[C@@H]1O)N1C=NC2=CC(Cl)=C(Cl)C=C12
Doramapimod	CC1=CC=C(C=C1)N1N=C(C=C1NC(=O)NC1=CC=C(OCCN2CCOCC2)C2=C1C=CC=C2)C(C)(C)C
Rebastinib	CNC(=O)C1=CC(OC2=CC=C(NC(=O)NC3=CC(=NN3C3=CC4=CC=CN=C4C=C3)C(C)(C)C)C(F)=C2)=CC=N1
Sorafenib	CNC(=O)C1=NC=CC(OC2=CC=C(NC(=O)NC3=CC(=C(Cl)C=C3)C(F)(F)F)C=C2)=C1
Tanzisertib	C1COC[C@H]1N2C3=NC(=NC=C3N=C2NC4=C(C=C(C=C4F)F)F)NC5CCC(CC5)O
Bosutinib	COC1=CC(NC2=C(C=NC3=CC(OCCCN4CCN(C)CC4)=C(OC)C=C23)C#N)=C(Cl)C=C1Cl
Mirdametinib	C1=CC(=C(C=C1I)F)NC2=C(C=CC(=C2F)F)C(=O)NOC[C@@H](CO)O
Paroxetine	FC1=CC=C(C=C1)[C@@H]1CCNC[C@H]1COC1=CC2=C(OCO2)C=C1
PP-121	NC1=NC=NC2=C1C(=NN2C1CCCC1)C1=CC2=C(NC=C2)N=C1
Sorafenib	CNC(=O)C1=NC=CC(OC2=CC=C(NC(=O)NC3=CC(=C(Cl)C=C3)C(F)(F)F)C=C2)=C1
Staurosporine	CC12C(C(CC(O1)N3C4=CC=CC=C4C5=C6C(=C7C8=CC=CC=C8N2C7=C53)CNC6=O)NC)OC

Table S2. List of test set compounds.

Test Compound	SMILES
Aumolertinib	CN(C)CCN(C)C1=CC(=C(C=C1NC(=O)C=C)NC2=NC=CC(=N2)C3=CN(C4=CC=CC=C43)C5CC5)OC
Rociletinib	CC(=O)N1CCN(CC1)C2=CC(=C(C=C2)NC3=NC=C(C(=N3)NC4=CC(=CC=C4)NC(=O)C=C)C(F)(F)F)OC
Olmutinib	CN1CCN(CC1)C2=CC=C(C=C2)NC3=NC4=C(C(=N3)OC5=CC=CC(=C5)NC(=O)C=C)SC=C4
Erlotinib	COCCOC1=C(C=C2C(=C1)C(=NC=N2)NC3=CC=CC(=C3)C#C)OCCOC
Fruquintinib	CC1=C(C2=C(O1)C=C(C=C2)OC3=NC=NC4=CC(=C(C=C43)OC)OC)C(=O)NC
Axitinib	CNC(=O)C1=CC=CC=C1SC2=CC3=C(C=C2)C(=NN3)/C=C/C4=CC=CC=N4
Cabozantinib	COC1=CC2=C(C=CN=C2C=C1OC)OC3=CC=C(C=C3)NC(=O)C4(CC4)C(=O)NC5=CC=C(C=C5)F
Repotrectinib	CC12CCC(CC1)C3(C2)C(=O)N(C=C3)C4=NC=C(C=C4)C5=C(C=C(C=C5)F)F
Ensartinib	C[C@@H]1CN(C[C@@H](N1)C)C(=O)C2=CC=C(C=C2)NC(=O)C3=NN=C(C(=C3)O[C@H](C)C4=C(C=CC(=C4Cl)F)Cl)N
Lazertinib	CN(C)C(=O)C=CC(=O)NC1=C(C=C(C=C1)NC2=NC=C(C=C2)C3=CN=C4N3C=C(N4)C(C)C)OC
Zongertinib	CN(C)C(=O)C=CC(=O)NC1=C(OC)C=C(C=C1)NC2=NC=C(C=C2)C3=CN=C4N3C=C(N4)C(C)C
Inavolisib	CC1(C(N(C2=C(N1)C=NC(=N2)N3CCOCC3)C4=CC=C(C=C4)F)C5=CC=C(C=C5)C(=O)N)OC
Sunvozertinib	CC(C)(C1=CC(=C(C=C1NC2=NC(=NC=C2)NC3=C(C=C(C(=C3)NC(=O)C=C)N4CC[C@H](C4)N(C)C)OC)Cl)F)O
Asciminib	C1CN(C[C@@H]1O)C2=C(C=C(C=N2)C(=O)NC3=CC=C(C=C3)OC(F)(F)Cl)C4=CC=NN4
Selpercatinib	CC(C)(COC1=CN2C(=C(C=N2)C#N)C(=C1)C3=CN=C(C=C3)N4CC5CC(C4)N5CC6=CN=C(C=C6)OC)O
Quizartinib	CC(C)(C)C1=CC(=NO1)NC(=O)NC2=CC=C(C=C2)C3=CN4C5=C(C=C(C=C5)OCCN6CCOCC6)SC4=N3
Pacritinib	C1CCN(C1)CCOC2=C3COC/C=C/COCC4=CC(=CC=C4)C5=NC(=NC=C5)NC(=C3)C=C2
Dabrafenib	CC(C)(C)C1=NC(=C(S1)C2=NC(=NC=C2)N)C3=C(C(=CC=C3)NS(=O)(=O)C4=C(C=CC=C4F)F)F
Selumetinib	CN1C=NC2=C1C=C(C(=C2F)NC3=C(C=C(C=C3)Br)Cl)C(=O)NOCCO
Pralsetinib	CC1=CC(=NN1)NC2=NC(=NC(=C2)C)C3CCC(CC3)(C(=O)N[C@@H](C)C4=CN=C(C=C4)N5C=C(C=N5)F)OC
Lorlatinib	C[C@@H]1C2=C(C=CC(=C2)F)C(=O)N(CC3=NN(C(=C3C4=CC(=C(N=C4)N)O1)C#N)C)C
Midostaurin	C[C@@]12[C@@H]([C@@H](C[C@@H](O1)N3C4=CC=CC=C4C5=C6C(=C7C8=CC=CC=C8N2C7=C53)CNC6=O)N(C)C(=O)C9=CC=CC=C9)OC
Cobimetinib	C1CCN[C@@H](C1)C2(CN(C2)C(=O)C3=C(C(=C(C=C3)F)F)NC4=C(C=C(C=C4)I)F)O
Zasocitinib	CNC1=CC(=NC2=C(C=NN12)C(=O)N[C@@H]3CC[C@H]3OC)NC4=CC=CN(C4=O)C5=CC=CC=N5
Delgocitinib	C[C@H]1CN([C@]12CCN(C2)C3=NC=NC4=C3C=CN4)C(=O)CC#N
Foretinib	COC1=CC2=C(C=CN=C2C=C1OCCCN3CCOCC3)OC4=C(C=C(C=C4)NC(=O)C5(CC5)C(=O)NC6=CC=C(C=C6)F)F
Tovorafenib	C[C@H](C1=NC=C(S1)C(=O)NC2=NC=C(C(=C2)C(F)(F)F)Cl)NC(=O)C3=C(C(=NC=N3)N)Cl
Tucatinib	CC1=C(C=CC(=C1)NC2=NC=NC3=C2C=C(C=C3)NC4=NC(CO4)(C)C)OC5=CC6=NC=NN6C=C5
Dacomitinib	COC1=C(C=C2C(=C1)N=CN=C2NC3=CC(=C(C=C3)F)Cl)NC(=O)/C=C/CN4CCCCC4
Avapritinib	C[C@](C1=CC=C(C=C1)F)(C2=CN=C(N=C2)N3CCN(CC3)C4=NC=NN5C4=CC(=C5)C6=CN(N=C6)C)N
Tepotinib	CN1CCC(CC1)COC2=CN=C(N=C2)C3=CC=CC(=C3)CN4C(=O)C=CC(=N4)C5=CC=CC(=C5)C#N
Umbralisib	C[C@@H](C1=C(C(=O)C2=C(O1)C=CC(=C2)F)C3=CC(=CC=C3)F)N4C5=NC=NC(=C5C(=N4)C6=CC(=C(C=C6)OC(C)C)F)N
Capivasertib	C1CN(CCC1(C(=O)N[C@@H](CCO)C2=CC=C(C=C2)Cl)N)C3=NC=NC4=C3C=CN4
Capmatinib	CNC(=O)C1=C(C=C(C=C1)C2=NN3C(=CN=C3N=C2)CC4=CC5=C(C=C4)N=CC=C5)F
Ripretinib	CCN1C2=CC(=NC=C2C=C(C1=O)C3=CC(=C(C=C3Br)F)NC(=O)NC4=CC=CC=C4)NC

Figure S1. Compound test-reference assigned clusters for kinase transitive mapping.

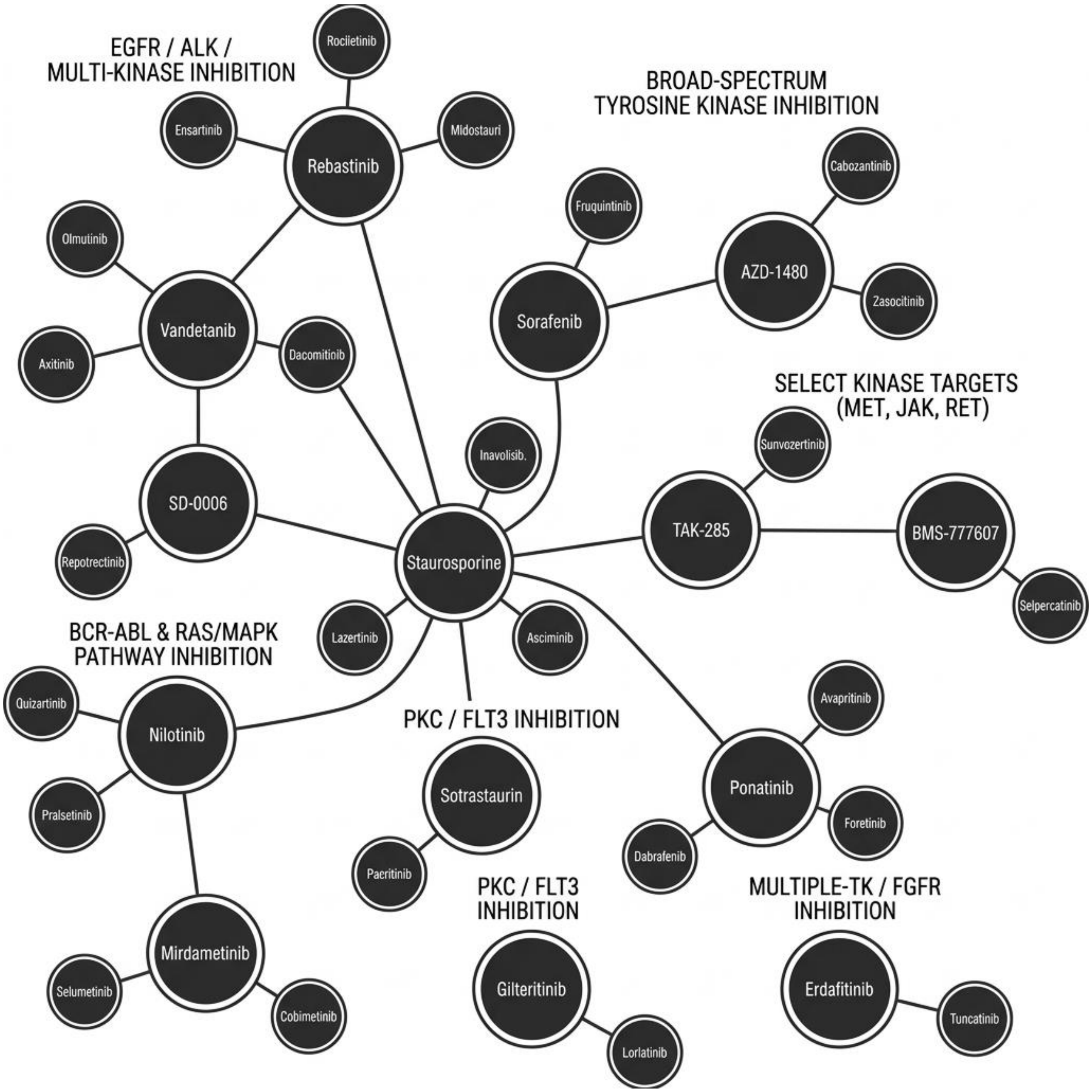

Table S3. Estimated pIC50 values for test set reference-target assignment per test compound

Trial_ID	Drug_Name	Assigned_Kinase	Database_pIC50	Source
**1**	Vandetanib	EGFR	7.3	ChEMBL
**2**	Sorafenib	VEGFR2	7.8	BindingDB
**3**	Vandetanib	VEGFR2	8.1	ChEMBL
**4**	AZD-1480	RET	8.0	PubChem
**5**	SD-0006	ROS1	7.5	BindingDB
**6**	Staurosporine	EGFR	9.0	ChEMBL
**7**	TAK-285	HER2	7.8	ChEMBL
**8**	Staurosporine	BCR-ABL	9.0	BindingDB
**9**	Mirdametinib	MEK1	9.5	ChEMBL
**10**	Nilotinib	RET	7.5	PubChem
**11**	Gilteritinib	FLT3	9.5	ChEMBL
**12**	Gilteritinib	ALK	7.8	ChEMBL
**13**	Rebastinib	FLT3	8.7	BindingDB
**14**	Mirdametinib	MEK2	9.5	ChEMBL
**15**	AZD-1480	JAK2	9.6	BindingDB
**16**	Ponatinib	VEGFR2	8.0	ChEMBL
**17**	Vandetanib	HER2	7.0	ChEMBL
**18**	Ponatinib	KIT	8.5	BindingDB
**Average**			8.3	
**19**	BMS-777607	FLT3	6.0	BindingDB
**20**	Nilotinib	FLT3	6.8	ChEMBL
**21**	Rebastinib	MET	6.5	BindingDB
**22**	Sotrastaurin	JAK2	6.5	PubChem
**23**	Ponatinib	BRAF	6.8	ChEMBL
**24**	Erdafitinib	KIT	5.5	BindingDB
**25**	Staurosporine	PI3Ka	6.0	ChEMBL
**26-35**	Multiple	Various	<6.5	Multiple
